# Incidence of periprosthetic joint infection after primary total hip arthroplasty is underestimated: a synthesis of meta-analysis and bibliometric analysis

**DOI:** 10.1186/s13018-023-04060-5

**Published:** 2023-08-21

**Authors:** Zi-Jun Zeng, Fang-Ming Yao, Wei He, Qiu-Shi Wei, Min-Cong He

**Affiliations:** 1 The Third School of Clinical Medicine, Guangzhou University of Chinese Medicine, Guangzhou, China; 2 Guangdong Research Institute for Orthopedics and Traumatology of Chinese Medicine, Guangzhou, China; 3 The Third Affiliated Hospital of Guangzhou University of Chinese Medicine, Guangzhou, China

**Keywords:** Periprosthetic joint infection, PJI, Arthroplasty, THA, Incidence, Epidemiology, Bibliometrics, Meta-analysis

## Abstract

The American Musculoskeletal Society updated the diagnostic criteria for periprosthetic joint infection (PJI) in 2011 and 2018. However, the overall incidence of PJI since the introduction of these new standards has not been assessed. In order to fill this knowledge gap, a single-group meta-analysis was conducted using articles obtained from several databases, focusing on the incidence of PJI after primary total hip arthroplasty (THA). Our study revealed a significant difference in the incidence of PJI reported by different national or regional databases. Moreover, most cases of PJI were found to be underestimated. This highlights the crucial need for standardized diagnostic criteria and monitoring methods to accurately identify and track cases of PJI. Furthermore, a bibliometric analysis was conducted to provide a comprehensive overview of the current state of research on PJI after THA. This analysis explored the most productive countries, organizations, journals, and individuals in this research area. Additionally, it identified the research trends and hotspots of the last decade, highlighting the advancements and areas of focus in this field. By conducting these analyses, the study aims to contribute to the understanding of PJI after THA and provide valuable insights for clinicians, researchers, and policymakers involved in the management of this condition.

**Supplementary Information:**

The online version contains supplementary material available at 10.1186/s13018-023-04060-5.

## Introduction

Arthroplasty has been clinically proven to be an effective surgical procedure for the relief of joint pain, and more and more patients are willing to undergo surgical treatment. The number of postoperative complications has also increased, with periprosthetic joint infection (PJI) being one of the most important complications after arthroplasty. The incidence of PJI is gradually gaining attention.

Before the development of the current diagnostic criteria for PJI, the most widely used worldwide, which was revised by the American Musculoskeletal Infection Society in 2011, the previous diagnostic criteria [1] for PJI were unclear and confusing. In a case-review study, Javad et al. [2] found great variation in diagnostic sensitivity among different diagnostic criteria. This makes the study of PJI with various definitions of infection very complicated. But this most widely used PJI diagnosis also suffers from a lack of sensitivity. In 2018, new diagnostic criteria [3] for PJI were proposed at the 2nd International Consensus Meeting. Haitao [4] compared the new 2018 diagnostic criteria to 2011 and found that the sensitivity and specificity of the diagnosis had improved. Although Bennie [5] had already done a META analysis study in 2014 on the incidence of infection in primary total hip arthroplasty (THA), the medical records included in their study were too early. Most of these studies were published before 2011 and required more consistency in their diagnostic criteria. Therefore, an updated study of the incidence of PJI is warranted. Meta-analysis is a powerful method of research synthesis that allows researchers to systematically analyze and combine data from multiple studies. It is an important tool for evaluating the current state of research and identifying trends and patterns across different studies. In particular, meta-analysis can be used to assess the effectiveness of interventions, examine the relationship between variables, and identify potential sources of bias or heterogeneity in a body of literature. By combining data from multiple studies, meta-analysis can provide a more comprehensive and reliable picture of a particular research question or topic. Bibliometric analysis is a valuable method of evaluation that can reveal the current state and direction of research [6]. In the first stage, we conducted a meta-analysis of incidence, which encompassed the literature from 2011 onwards, to obtain a thorough comprehension of the occurrence of PJI. In the second stage, we carried out a bibliometric analysis of the literature in the field since 2011 to aid in understanding the advancements made in PJI research and to facilitate future research studies.

## Method

### Meta-analysis

#### Search strategy

The systematic review described in this study was registered in the Prospero database under the registration number CRD42022383290. We used three electronic medical databases (Pubmed, Embase, and Web of Science) to search from their inception to October 2022. The searches were conducted in accordance with the PRISMA (Preferred Reporting Items for Systematic Reviews and Meta-Analyses) guidelines. The search terms included “(infection or infections or PJI) and (prevalence or incidence or epidemiology or morbidity) and (THA or hip or arthroplasty) and (peri-prosthetic or periprosthetic or Implant or prosthetic or prosthesis or prosthesis-related or periprosthetic)” (the search strategy is provided in Additional file 1: Table 1). No language restrictions were imposed during the search, and the final search was conducted on November 21, 2022.

#### Inclusion and exclusion criteria

We predefined the inclusion criteria for eligible studies as follows: (1) The study population was all-age patients after primary THA; (2) There were no restrictions regarding publication year. If more than 1 study evaluated the same population, only the larger study was included.;(3) The outcome was the point incidence of PJI after primary THA or sufficient data to calculate PJI among the general population or among clinical patients. Studies were excluded if (1) they were review articles, case reports, protocols, short communications, personal opinions, letters, posters, conference abstracts, or laboratory research; (2) they did not report sufficient data or unavailable relevant data for analysis, and efforts to contact the authors were unsuccessful; (3) they did not involve patients with PJI.

We additionally excluded two articles in the screening process, one because it only counted patients with short-stem THA [7], and the other because we thought the statistical method it used might lead to large errors [8].

#### Data extraction

Articles have been proposed according to title and abstract. The relevant abstracts and any articles without abstracts were selected for full-text review. Two researchers gathered information from the independently included studies and double-checked the information to ensure the integrity of the content. In case of disagreement, a third researcher was consulted to discuss the decision. We inserted the data into 2 tables, with one listing the database-based studies (Table 1) and the other listing the clinic-based studies (Table 2). The gathered data included first author, publication time, continent, country or region, the gender distribution of the sample, search criteria for database study, diagnostic criteria for clinical studies, age range, time to onset of PJI after THA, sample size, and incidence of PJI.

**Table 1:** Characteristics of the database-based studies of PJI

study	PJI	Primary THA	Incidence (%)	Surgery time (THA)	Infection time	Search criteria
Muscatelli et al. [19]	435	25,281 female:25,281 male:21,118	0.94	2015/10/1–2018/9/30	90 days 90-365 days	Non-refurbished database(ICD-9)
Jin et al. [13]	1190	109,412	1.09	2002/7/1–2017/12/31	90 days	Non-refurbished database(T84.5)
Bae et al. [14]	16 female:7 male:9	4758	0.33	2003/1–2017/12	NA	Refurbishment database
Bulow et al. [11]	2173 female:1062 male:1111	88,830 female:50,151 male:38,679	2.45	2008–2015	90 days	Non-refurbished database(ICD-10)
Bulow et al. [11]	410 female:209 male:201	18,854 female:10,915 male:7939	2.17	2016–2018	90 days	Non-refurbished database(ICD-10)
Bozzo et al. [20]	1034	100,674 female:59,237 male:41,437	1.03	2002/1/1–2016/12/31	1 years 2 years	Non-refurbished database(ICD-10 CA)
Kurtz et al. [26]	12,397 female:7117 male:5280	1,158,742 female:726,354 male:432,388	1.07	2005/1/1–2015/9/30	NA	Refurbishment database(ICD-9 CM)
Gundtoft et al. [21]	271	48,867 female:55.1%	0.55	2005–2015	1 years	Refurbishment database(ICD-10) + Intraoperative bacterial culture
Jung et al. [22]	350	33,337	1.05	2014/5–2018/1	NA	Refurbishment database
Grammatico-Guillon et al. [15]	383	21,633	1.77	2008–2012	NA	Non-refurbished database(ICD-10)
Dale et al. [12]	1715	261,103	0.66	1995–2004	0–3 months 3–12 months 1–2 years	Refurbishment database

Study	Study design	Region	Primary THA Age(years)	PJI Age(years)
Muscatelliv et al. [19]	Retrospective	USA(North America)	≥ 65: 24,510	NA
Jin et al. [13]	Retrospective	Australia (Oceania)	NA	NA
Bae et al. [14]	Retrospective	Korea (Asia)	Average 62.8(± 27.4)	40–49:2 50–59:2 60–69:4 70–79:3 80–89:2 90–99:3
Bulow et al. [11]	Retrospective	Sweden (Europe)	< 50: 4645 50–60: 11,812 60–70: 28,981 70–80: 30,386 > 80: 13,006	< 50:104 50–60:231 60–70:609 70–80:776 > 80:453
Bulow et al. [11]	Retrospective	Denmark (Europe)	< 50: 880 50–60: 2460 60–70: 5226 70–80: 7201 > 80: 3087	< 50:9 50–60:45 60–70:112 70–80:164 > 80:80
Bozzo et al. [20]	Retrospective	Canada (North America)	55–69: 49,812 70–84: 46,378 ≥ 85: 484	NA
Kurtz et al. [26]	Retrospective	USA(North America)	65–69: 310,721 70–74: 303,952 75–79: 263,128 80–84: 182,096 > 85: 98,845	65–69:3711 70–74:3360 75–79:2796 80–84:1719 > 85:811
Gundtoft et al. [21]	Retrospective	Denmark (Europe)	68.85	NA
Jung et al. [22]	Retrospective	Finland (Europe)	NA	NA
Grammatico-Guillon et al. [15]	A Cohort Study	France (Europe)	NA	NA
Dale et al. [12]	Retrospective	Norway (Europe)	< 40: 3 40–59: 35 60–69: 58 70–79: 75 80–89: 2	NA

**Table 2:** Characteristics of the clinic-based studies of PJI

Study	PJI	Primary THA	Incidence (%)	Surgery time (THA)	Follow-up time	Diagnostic criteria
Tella et al. [16]	4 female:3 male:1	583 female:320 male:263	0.69	2016	Average 595 days	NA
Yildiz et al. [17]	21	1260 female:926 male:334	1.67	2016/1–2019/12	> 90 days	MSIS
Aggarwal et al. [23]	60	3574 female:2003 male:1571	1.68	2011–2016	Average 3.72 years	MSIS
Franco-Cendejas et al. [24]	4	179 female:114 male:65	2.23	2011/8–2012/7	Average 402 days	MSIS
Alp [18]	5	415	1.2	2011/4–2013/4	NA	Local CDC standards, approximate to MSIS
Renaud et al. [25]	49	2403	2.04	1990–2007	NA	Arthrobacter culture

Study	Study design	Region	Primary THA age (years)	PJI age (years)
Tella et al. [16]	Retrospective	Italy	Average 62.2	Average 62.8
Yildiz et al. [17]	Retrospective	Turkey	NA	NA
Aggarwal et al. [23]	Retrospective	USA	Average 63	NA
Franco-Cendejas et al. [24]	Prospective	Mexico	NA	NA
Alp et al. [18]	Retrospective	Turkey	NA	NA
Renaud et al. [25]	Retrospective	Canada	NA	NA

#### Quality assessment

In our study, we utilized the JBI Independent Assessment Scale to evaluate the quality of the articles we obtained. This tool is specifically designed to assess the quality of systematic reviews and meta-analyses, including an assessment of the included studies, data extraction and synthesis, as well as the presentation and interpretation of results. Its purpose is to aid readers in determining the credibility and applicability of a given systematic review or meta-analysis. The scale was developed by the Joanna Briggs Institute, an internationally recognized research institute based in Australia. Overall, this scale is an important resource for evaluating the quality of systematic reviews and meta-analyses. Additional file 1: Table 2 displays the assessment scores we assigned to the articles based on our evaluation of the provided questions. A higher total score indicates a higher quality of the study and a lower risk of bias. We categorized the studies into three groups: high quality (scores below 49%), moderate quality (scores between 50 and 69%), and low quality (scores above 70%), based on the percentage of positive responses.

#### Data analysis

Data analysis was performed using the Meta modules in the R statistical package, version 4.0.5. We adopted the logit method to transform the incidence of PJI reported in each literature. Then, We did the Shapiro-Wilk normality test and obtained the pooled incidence (with 95% CIs) of PJI. We assessed the heterogeneity of the combined results for the incidence of PJI by using the Q statistic and the *I*^2^ statistic [9, 10]. In general, when *P* > 0.05 (for *Q* statistic) and *I*^2^ < 50%, the combined result is statistically homogeneous and a fixed-effects model can be used; when *P* > 0.05 and *I*^2^ > 50%, it indicates statistical heterogeneity and a random-effects model should be used. Forest plots were used to graphically display the incidence of overall and subgroup. We used Egger test to assess publication bias and show it using funnel plots. The trim and fill method was used to evaluate the effect of publication bias on the results when publication bias was present. We also used meta-regression analysis to assess heterogeneity between studies. In addition, subgroup analysis by publication time, continent, sex, Search criteria for database study, age range, and time to onset of PJI after THA.

### Bibliometric analysis

#### Data acquisition and search strategy

In this study, we utilized the Web of Science Core Collection (WOSCC) database as our primary source of information. On March 3, 2023, we conducted a search using a predefined strategy: (TS = (total hip arthroplasty)) and TS = (infection). We did not impose any language restrictions on the publications. To ensure the quality and relevance of the articles, we excluded several types of material, such as editorial material, letters, conference abstracts, revisions, conference proceedings papers, book chapters, and retracted publications.

#### Data analysis

Data was collected from 4026 articles and analyzed using three bibliometric tools: R version 4.2.0, VOSviewer, and CiteSpace. The R package Bibliometrix was used to process the dataset and generate various bibliometric indicators and visualizations. This package provides a set of functions for importing, managing, analyzing, and visualizing bibliometric data.

VOSviewer is a software tool that enables the construction and visualization of bibliometric networks. These networks can include various entities such as journals, researchers, or individual publications and can be constructed based on various relations including citation, bibliographic coupling, co-citation, or co-authorship. Additionally, VOSviewer offers text mining functionality that can be utilized to analyze the content of a large corpus of scientific literature.

The results generated by VOSviewer provide valuable insights into the structure and relationships within a scientific field. For instance, a co-authorship network can reveal patterns of collaboration among researchers, while a citation network can highlight the most influential publications within a field. CiteSpace was used for burst analysis to identify significant burst terms and their time span in the field of THA and infection. This Java-based software application is designed for visualizing and analyzing trends and patterns in scientific literature.

## Result

### Meta-analysis

#### Literature search and included studies

**Fig. 1:**
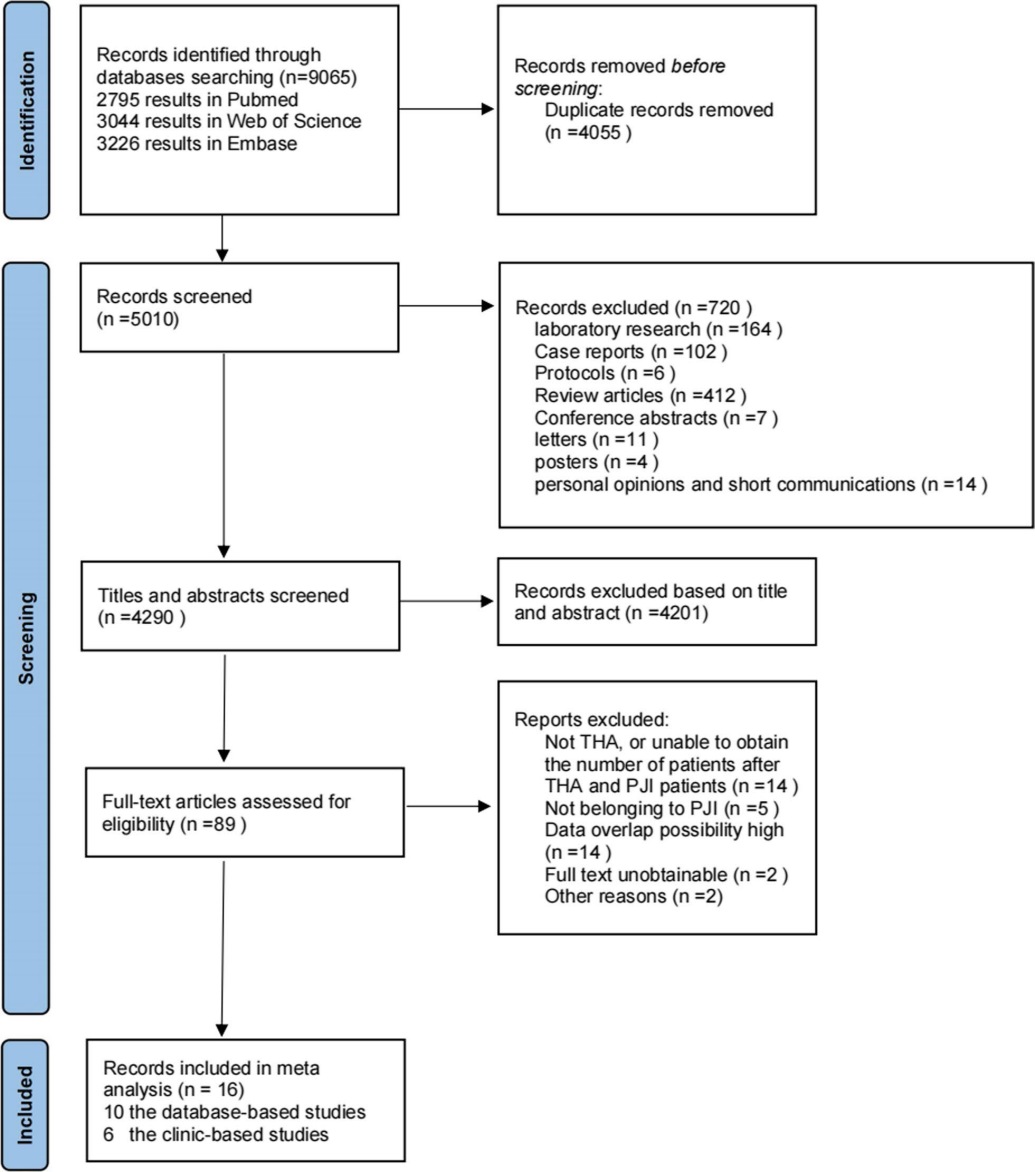
Candidate study selection workflow for meta-analysis

Using the search term “(infection or infections or PJI) and (prevalence or incidence or epidemiology or morbidity) and (THA or hip or arthroplasty) and (peri-prosthetic or periprosthetic or Implant or prosthetic or prosthesis or prosthesis-related or periprosthetic)” resulted in 9065 records from the 3 databases (2795 results in Pubmed; 3044 results in Web of Science; 3226 results in Embase). After duplicate removal, our literature searches yielded 5010 articles. We screened 4290 potentially relevant reports, reviewed 89 articles in full-text, and finally included 16 studies in the analysis. Details of the inclusion process are shown in Fig. 1. The characteristics of 10 database-based studies are summarized in Table 1 and 6 clinic-based studies are in Table 2.

The 10 database-based studies, with a combined population of 1,892609, cover 4 continents (North America, Oceania, Europe, and Asia) and 9 countries (including the USA, Australia, Korea, Sweden, Denmark, Canada, Finland, France, and Norway). In addition, because Bulow [11] grouped the populations from Sweden and Denmark separately, we conducted the two data sources in this article in separate Table 1. For Dale. H’s [12] study, we only included data from this study from 1995 to 2004 because of possible overlap with previous studies.

The 6 clinical-based studies, with a combined population of 8419, covered 4 continents (North America, South America, Europe, and Asia) and 5 countries (including Italy, Turkey, USA, Mexico, and Canada).

The JBI quality assessment showed that 6 [13–18] studies had a high risk bias, 8 [12, 19–25] studies had a moderate risk bias, and 2 [14, 26] studies had a low risk bias. The whole process was done by two researchers. A third researcher decides when disagreements arise.

#### Overall incidence of PJI

**Fig. 2:**
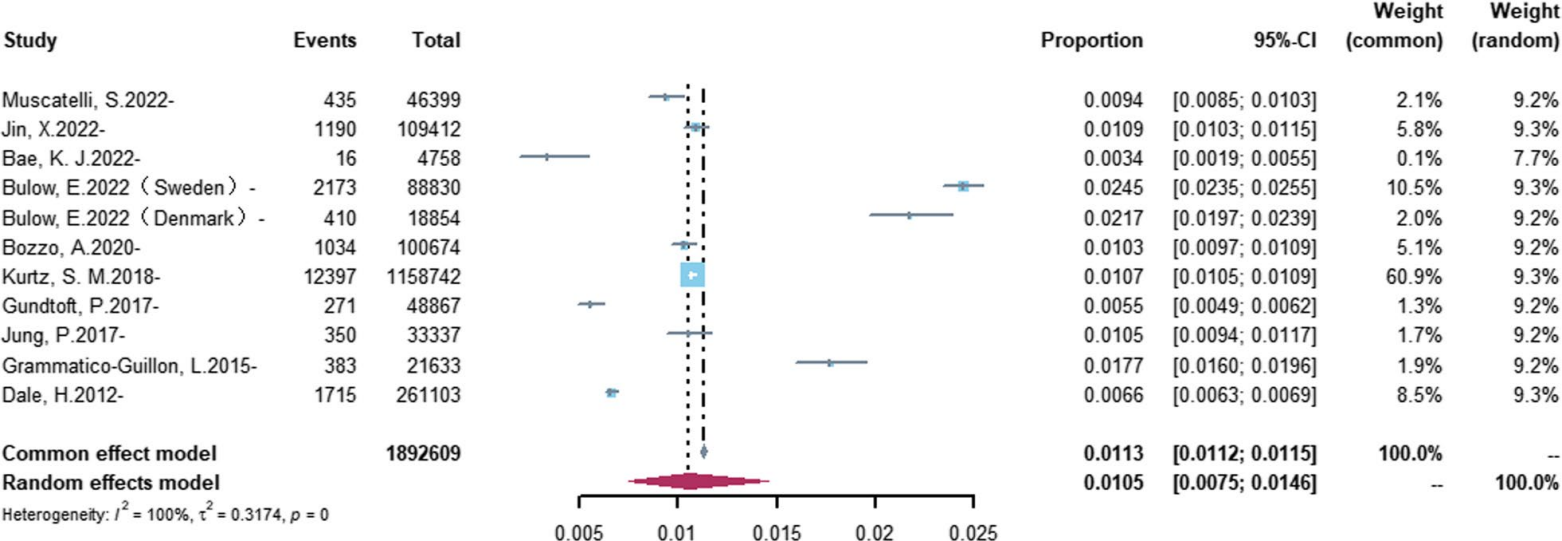
Forest plot of the overall incidence of PJI in the database-based studies. The size of the squares represents the proportion (95% CI) for each of the studies. The size of the diamonds represent the overall proportion (95% CI)

**Fig. 3:**
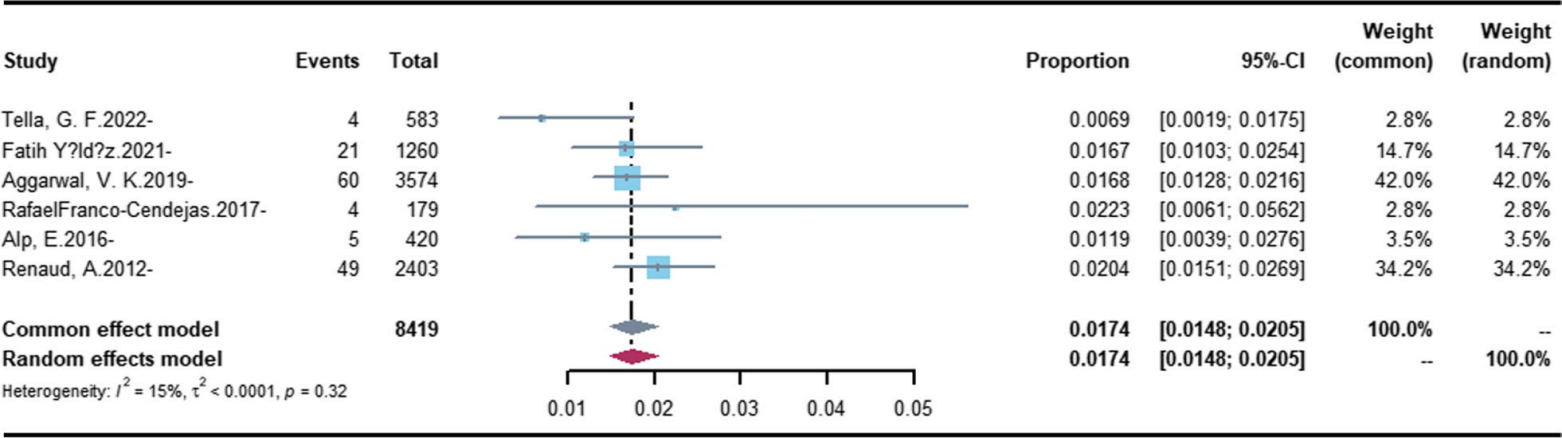
Forest plot of the overall incidence of PJI in the clinic-based studies. The size of the squares represents the proportion (95% CI) for each of the studies. The size of the diamonds represent the overall proportion (95% CI)

Incidence estimates for PJI derived by meta-analysis are shown in Figs. 2 and 3. Estimates for the medical database-base studies [11–15, 19–22, 26] ranged from 0.34 to 2.45% (Fig. 2), and the random-effects overall pooled estimated incidence of PJI was 1.05% (95% CI 0.75–1.46%), with very high heterogeneity (*I*^2^ = 99.6%; heterogeneity test *P* = 0). Estimates for the clinic-based studies [16–18, 23–25] ranged from 0.69 to 2.23% (Fig. 3), and the common-effects pooled overall estimated incidence of PJI was 1.74% (95% CI 1.48–2.05%), with low heterogeneity. (*I*^2^ = 15.0%; heterogeneity test *P* = 0 0.32).

#### Incidence of PJI by search criteria

Among the studies based on medical databases, one was a study using patient information extracted from a database of joint prosthesis revision, with a total of 5 studies [12, 14, 21, 22, 26] (5 data sources, *n* = 1,506,807), and the combined incidence estimate of PJI was 1.00%( 95% CI 0.98–1.01%), with very high heterogeneity (*I*^2^ = 99.2%; heterogeneity test *P* < 0.01) (Additional file 1: Fig. 1). 5 studies [11, 13, 15, 19, 20] (6 data sources, *n* = 385,802) used patient information extracted from ICD code searches by regional statistical agencies of the medical nature, and the combined incidence estimate of PJI was 1.58% (95% CI 1.54–1.62%), also with very high heterogeneity (*I*^2^ = 99.5%; heterogeneity test *P* < 0.01) (Additional file 1: Fig. 1).

#### Incidence of PJI by age

Among the studies based on medical databases, 2 studies [11, 26](containing 3 data sources) in a total of 1,266,426 patients reported incidences of PJI after THA in patients ≥ 70 years or < 70 years of age(Overall age range of 50–80 years old). The results of these 2 studies indicated that in patients older than 70 years, the incidence of PJI was 1.90% (95% CI 1.02–3.51%; *I*^2^ = 99.2%; heterogeneity test *P* < 0.01) (Additional file 1: Fig. 2). In patients younger than 70 years of age, the incidence of PJI was 1.68% (95% CI 1.19–2.38%; *I*^2^ = 99.2%; heterogeneity test *P* < 0.01) (Additional file 1: Fig. 2). 2 studies [19, 20] reported the age of patients after THA only, and 1 study [14] reported the age of patients with PJI only. No studies of age stratification in clinic-based studies.

#### Incidence of PJI by sex

A sex difference in the incidence of PJI was observed between the 5 database-based studies [11, 14, 19, 20, 26]. 2 studies [19, 20] only reported the number of men and women after THA. 1 study [14] reported only the number of men and women with PJI. 2 [11, 26] of 5 database-based studies reported the number of men and women after THA (men = 479,006;women = 787,420) and the number of men and women with PJI. The pooled incidence of PJI was 1.58% (95% CI 0.98–2.54%) for women (*I*^2^ = 99.7%; heterogeneity test *P* < 0.01) and 2.07% (95% CI 1.22–3.48%) for men (I^2^ = 99.7%; heterogeneity test *P* < 0.01) (Additional file 1: Fig. 3). 3 clinic-based studies [16, 23, 24] reported sex differences in the incidence of PJI, but only 1 study [16] (women = 320; men = 263)reported the number of men and women after THA and PJI. The incidence of PJI was 0.94% for women and 0.38% for men.

#### Incidence of PJI by time after THA to PJI

The diagnostic criteria for PJI, developed by the American Musculoskeletal Infection Association in 2011 and revised at the first International Consensus Meeting (ICM) in 2013, define PJI as acute infection if it is less than 90 days and as chronic infection if it is more than 90 days. Among the studies based on databases, 6 studies [11–13, 19–21] differentiated the timing of PJI after THA, but one [20] of the studies did not report the exact number of people, so it could not be meta-combined. 3 [11, 13, 19] (*n* = 263,495) of 6 studies reported PJI occurred within 90 days after THA. The pooled incidence of PJI was 1.43% (95% CI 0.81–2.52%), with very high heterogeneity (*I*^2^ = 99.6%; heterogeneity test *P* < 0.01) (Additional file 1: Fig. 4). 3 [12, 19, 21] (*n* = 263,495) of 6 studies reported PJI occurred within 1 year after THA (Patients with PJI within 90 days excluded). The pooled incidence of PJI was 0.49% (95% CI 0.22–1.10%), with very high heterogeneity (*I*^2^ = 99.6%; heterogeneity test *P* < 0.01) (Additional file 1: Fig. 4). Information on the time of infection in the clinic-based studies is incomplete, so no statistics are available.

#### Incidence of PJI by publication time

The diagnostic criteria for PJI have changed again in 2018 since they were established in 2011, and to see if there was an association between the incidence of PJI and the time of publication, we performed an association analysis of the time of publication. The chart shows that the incidence of PJI fluctuated with time to release, ranging from 0.33 to 2.17% among the database-based studies (Table 1) and ranging from 0.69 to 2.23% among the clinic-based studies (Table 2). According to the scatterplot (Additional file 1: Figs. 5 and 6), the change in the incidence of PJI was not statistically significantly related to the time of publication. For further validation, we checked the time to publication and the incidence of PJI with Spearman rank correlation coefficient [27], and the results both were *P* > 0.05 (Additional file 1: Tables 3 and 4), with no statistically significant correlation between time to publication and the incidence of PJI.

#### Incidence of PJI by continent

Of the 10 [11–15, 19–22, 26] studies based on the database, there were 5 [11, 12, 15, 21, 22] studies from Europe, 3 [19, 20, 26] from North America, 1 [14] from Asia, and 1 [13] from Oceania. Among them, the incidence of PJI was 1.24% (95% CI 0.75–2.05%) in Europe (with very high heterogeneity [*I*^2^ = 99.8%; *P* = 0]), 1.02% (95% CI 0.95–1.10%) in North America (with very high heterogeneity [*I*^2^ = 77.0%; *P* = 0.01]), 0.34% in Asia and 1.09% in Oceania. (Additional file 1: Fig. 7) Of the 6 [16–18, 23–25] studies based on the clinic, there were 2 [17, 18] studies from Asia, 2 [23, 25] from North America, 1 [16] from Europe, and 1 [24] from South America. Among them, the incidence of PJI was 1.56% (95% CI 1.07–2.28%) in Asia (with low heterogeneity [*I*^2^ = 0%; *P* = 0.50]), 1.83% (95% CI 1.52–2.21%) in North America (with low heterogeneity [*I*^2^ = 3.7%; *P* = 0.31]), 0.69% in Europe and 2.23% in South America. (Additional file 1: Fig. 8).

#### Publication bias

Among the 10 [11–15, 19–22, 26] database-based studies, from the funnel plot it can be seen that the studies’ scatter distribution axis is not uniform on both sides (Additional file 1: Fig. 9), so we performed a trim and fill method and found that the heterogeneity results do not differ significantly (Additional file 1: Fig. 10), indicating that there is no significant publication bias. Further, Egger test suggested no statistically significant publication bias (*P* > 0.05) (Additional file 1: Fig. 11). Among the 6 [16–18, 23–25] clinic-based studies, The scatter points in the funnel plot are symmetrically distributed on both sides of the axis (Additional file 1: Fig. 12). Egger test suggested no statistically significant publication bias (*P* > 0.05) (Additional file 1: Fig. 13).

#### Sensitivity analyses

The heterogeneity of the studies we included was high. We conducted a study-by-study exclusion of individual studies for the 10 [11–15, 19–22, 26] database-based studies and the 6 [16–18, 23–25] clinic-based studies separately to observe the effect of individual studies on the combined effect. Both results showed no significant change in the effect size, indicating the good stability of the included studies. (Additional file 1: Figs. 14 and 15).

#### Meta-regression

There was high heterogeneity in the database-based studies, and we performed Meta single factor regression analysis on subgroups of sufficient studies. The results showed that, in database-based studies, search criteria were the sources of heterogeneity (*P* < 0.05), but the continent was not. (*P* > 0.05) (Additional file 1: Table 5).

### Bibliometric analysis

#### Basic data summary

In this study, a total of 4026 articles were analyzed. These articles were authored by a total of 14,517 researchers from 3670 research institutions across 82 countries. The articles were published in 492 different journals and had an average of 18.86 citations per article. According to Lotka’s Law [28], it was observed that authors who contributed to three studies accounted for 50% of the total number of authors.

#### Annual number of publications

Since 2011, there has been a rapid increase in the number of published articles on PJI after primary THA. In 2012, there were 233 articles published on this topic. By 2020, this number had grown to 465, representing an increase of more than 99%. However, the growth curve of the annual number of published articles on this topic has not been a continuous upward trend. In fact, there were years when the number of published articles decreased, such as in 2013 (− 5.58%), 2018 (− 5.23%), and 2022 (− 2.15%).

**Fig. 4:**
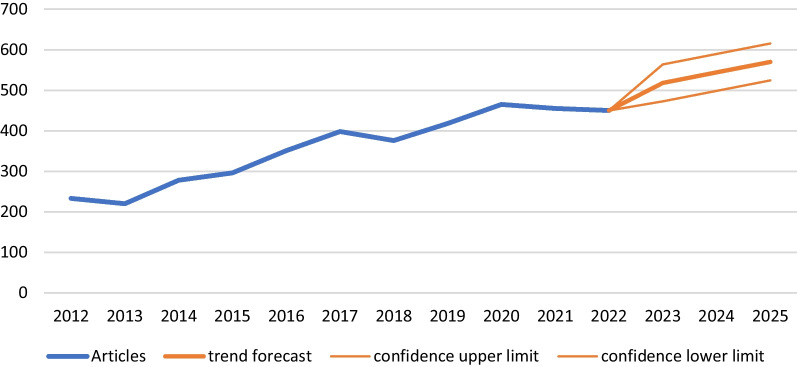
Annual publication numbers from 2012 to 2015

In order to analyze the future development trend of PJI after primary THA, we used the Holt-Winters method to analyze the data from 2012 to 2022 (excluding 2023 data because the year is not yet over). According to our analysis and the prediction curve shown in Fig. 4, we believe that the number of publications in this field will continue to increase annually. By 2025, we predict that the number of publications on PJI after primary THA will reach 570.

#### Analysis of the countries with the highest productivity

**Fig. 5:**
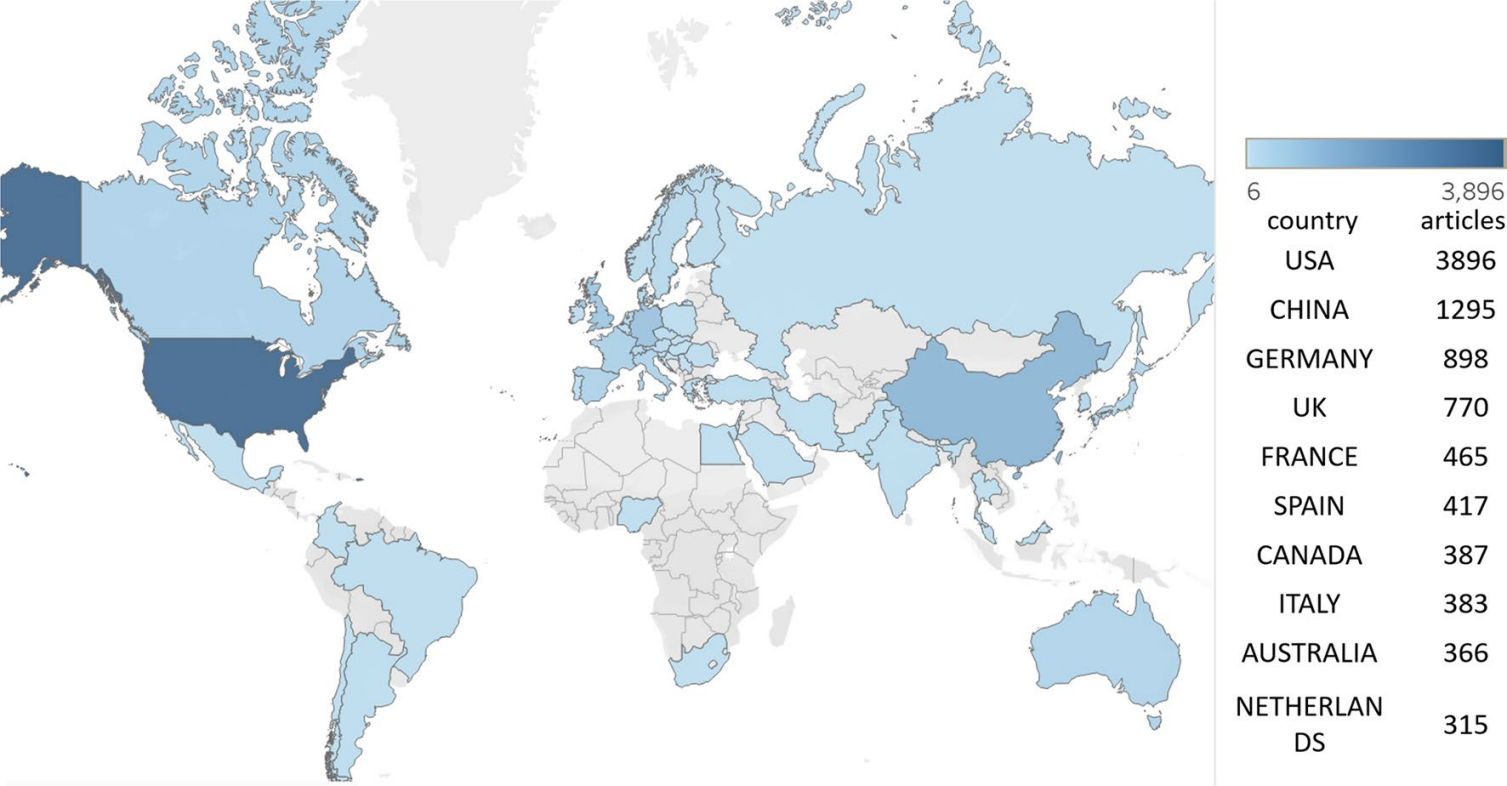
A global map showing the relative contribution of each country based on the number of publications

**Fig. 6:**
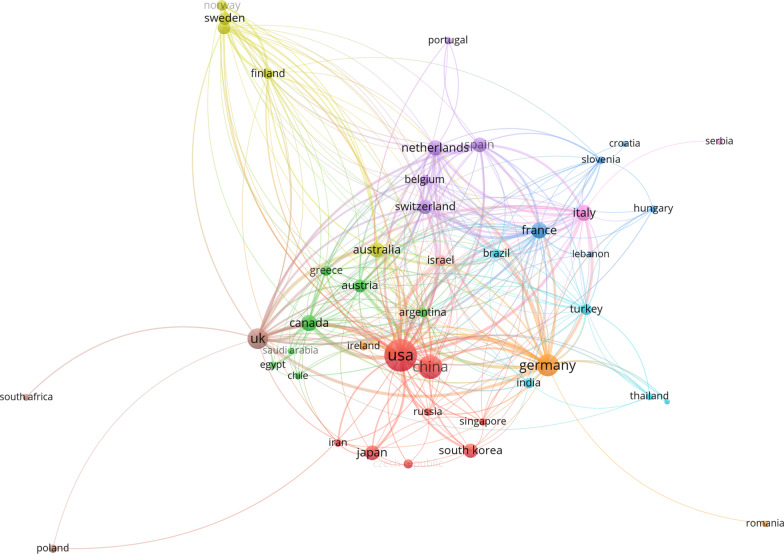
Network visualization of each country

Figure 5 shows the number of articles in each country. The USA has the largest number of publications (*n* = 3896), followed by China (*n* = 1295), Germany (*n* = 898), the UK (*n* = 770), France (*n* = 465) and Spain (*n* = 417). Less than 400 articles were published in each of the remaining countries. The majority of research collaborations involve the USA and other countries (Fig. 6). The top six collaborations are between the USA and China (*n* = 44), the USA and Israel (*n* = 35), the USA and Canada (*n* = 31), the USA and Germany (*n* = 31), the USA and the UK (*n* = 27), and Germany and Switzerland (*n* = 26).

#### Analysis of the most productive institutions

Additional file 1: Table 6 displays the top 10 most productive research institutions. Of these institutions, eight are located in the USA, one in China, and one in Canada. The Mayo Clinic in the United States experienced the largest increase in publications from 2020 to 2022, followed by Harvard Medical School in the United States and Hospital for Special Surgery.

#### Analysis of the most productive journals

Table 3 presents the top 10 journals with the highest number of publications. The annual number of publications in the journal can be seen in Fig. 4. Of these journals, four originate from the USA, three from the UK, two from Germany, and one from Sweden. According to the Journal Citation Reports (JCR), 50% of these journals are classified within the first quartile (Q1).

**Table 3:** The top 10 most cited publications

Publication	Total citations	Journal	IF	JCR
Osmon et al. [31]	1429	Clinical Infectious Diseases	20.99	Q1
Kurtz et al. [52]	1052	The Journal of Arthroplasty	4.435	Q1
Tande et al. [53]	851	Clinical microbiology reviews	50.129	Q1
Parvizi et al. [3]	730	The Journal of Arthroplasty	4.435	Q1
Goodman et al. [54]	549	Biomaterials	15.304	Q1
Bratzler et al. [30]	489	Surgical Infections	1.853	Q3
Raphel et al. [55]	443	Biomaterials	15.304	Q1
Kapadia et al. [29]	414	Lancet	202.731	Q1
Zmistowski et al. [56]	355	The Journal of Bone and Joint Surgery. American volume	6.558	Q1
Bozic et al. [57]	310	The Journal of Bone and Joint Surgery. American volume	6.558	Q1

Furthermore, an analysis was conducted on journals that published an average of more than 10 articles annually between 2020 and 2022. These include *Journal of Arthroplasty* (*n* = 63), *Bone & Joint Journal* (*n* = 10), *Hip International* (*n* = 12), *Journal of Bone and Joint Surgery-American Volume*, *Archives of Orthopaedic and Trauma Surgery* (*n* = 10), and *BMC Musculoskeletal Disorders* (*n* = 11).

#### Analysis of the most cited publications

Table 3 lists the total citations, journal name, impact factor (IF), and JCR ranking of several publications. The publication with the highest impact factor is “Kapadia. BH, 2016, *Lancet*” [29] with an impact factor of 202.731 and published in the Lancet with a JCR ranking of Q1. All the publications listed in the table are ranked Q1 except for “Bratzler. DW, 2013, *Surgical Infections*” [30] which is ranked Q3. There are two publications from J Arthroplasty and two publications from J Bone Joint Surg Am. The publication [31] with the highest number of citations is “Diagnosis and Management of Artificial Joint Infections: Clinical Practice Guidelines” with 1429 citations and published in Clinical Infectious Diseases with an impact factor of 20.99 and a JCR ranking of Q1. It provides evidence-based and opinion-based recommendations for the diagnosis and management of patients with PJI. The guidelines are intended for use by infectious disease specialists, orthopedists, and other healthcare professionals who care for patients with PJI.

#### Analysis of the most productive authors

**Fig. 7:**
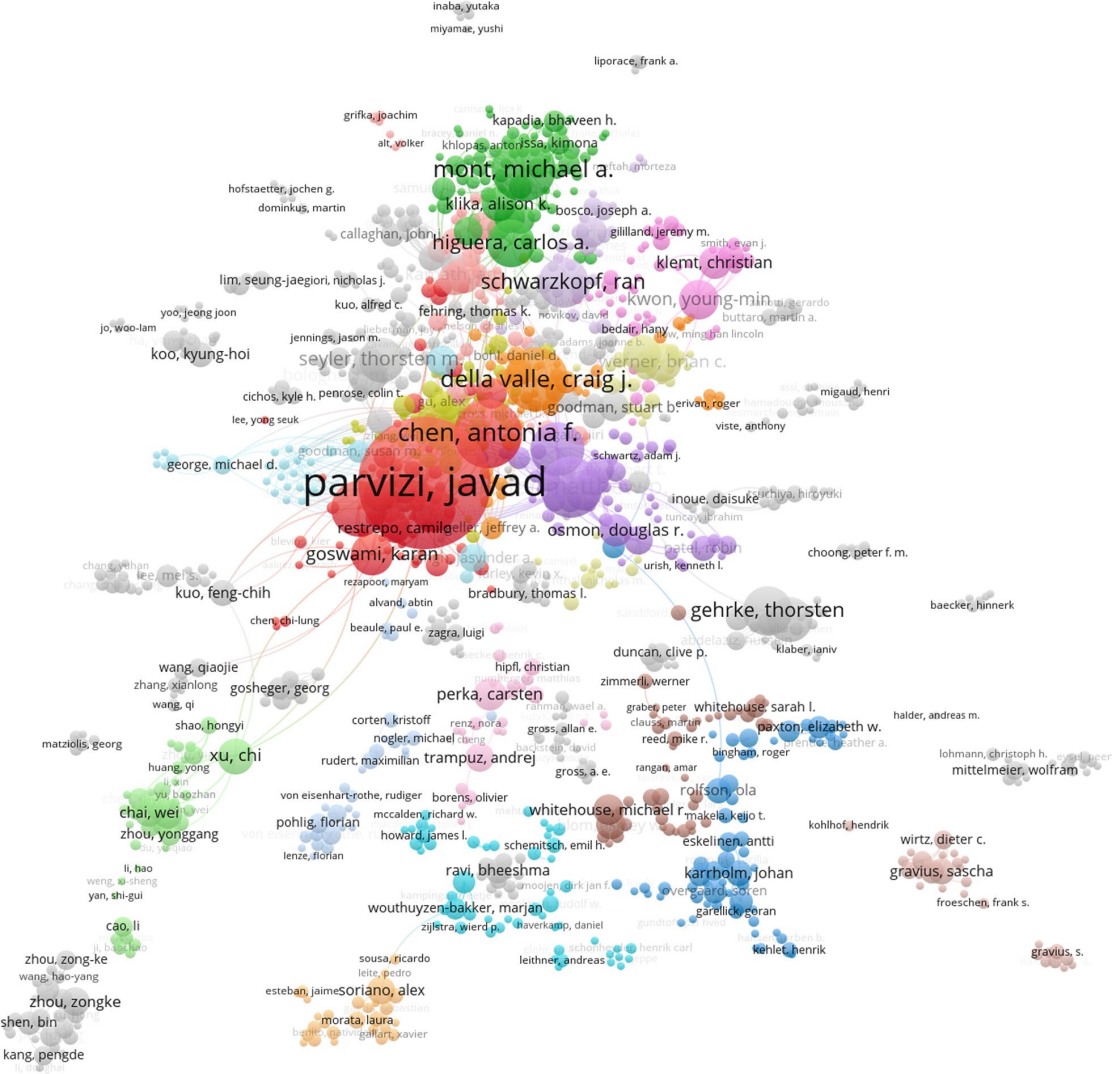
Network visualization of author

Additional file 1: Table 8 presents the top 10 authors with the highest number of publications. Javad Parvizi, MD, affiliated with the Rothman Institute at Thomas Jefferson University, has been identified as the most prolific author in the field, with a total of 170 publications and 7615 citations. Furthermore, as depicted in Fig. 7’s author collaboration network, there is frequent collaboration among most authors who have extensively studied PJI.

#### Analysis of keywords

Our search results yielded a total of 4957 author keywords. After merging keywords with the same meaning, we were left with 4896 unique keywords. We then filtered out keywords that appeared less than 20 times, resulting in 62 keywords that met our filtering criteria. We present the 50 most frequently occurring keywords in Additional file 1: Table 9.

**Fig. 8:**
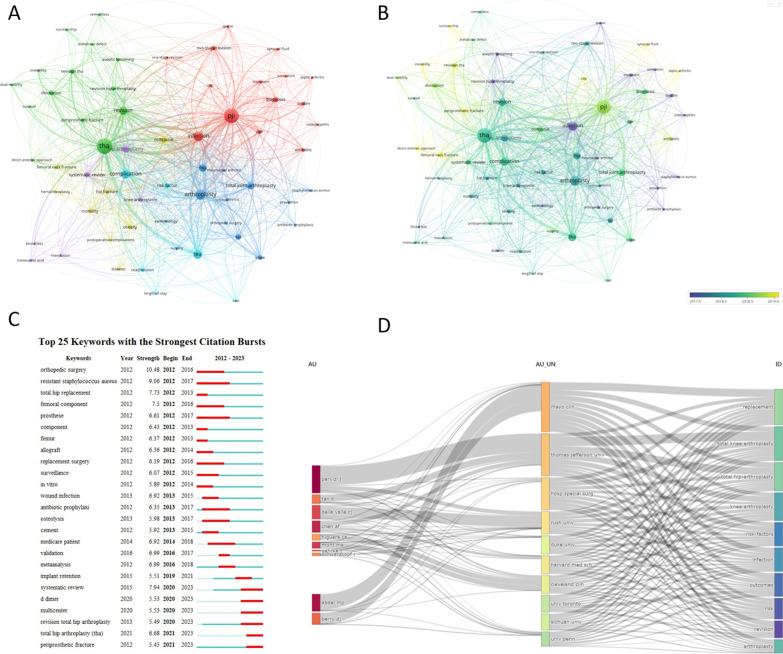
**A** network visualization of author keywords; **B**, overlay visualization of author keywords; **C**, visualization map of top 20 keywords with the strongest citations bursts; **D**, three-field plot of the Author Keywords analysis on PJI after primary THA Notes: three-field plot of the keywords plus analysis: (left field: authors; middle field: affiliations; right field: keywords plus)

We also performed a network visualization of these high-frequency keywords (Fig. 8A). The size of the nodes represents the frequency of the keywords, while the distance between two nodes represents the strength of their association. Keywords that were closer together were grouped into the same cluster, which broadly reflects the major themes in the field of PJI research after primary THA.

The first cluster is shown in red and mainly relates to diagnosis and treatment. The most common words include “PJI,” “infection,” “diagnosis,” “two-stage revision,” “DAIR,” “biofilm,” “antibiotic,” and “CRP.” The second cluster is shown in green and mainly relates to revision techniques. The most frequent words include “THA” and related words such as “dislocation” and “aseptic loosening.” The third cluster is shown in dark blue and mainly relates to total joint arthroplasty and the prevention of PJI, with frequent words including “arthroplasty,” “ hip,” “ total joint arthroplasty,” and “prevention.” The fourth cluster is shown in yellow and reflects content related to prognosis, with frequent words including “outcome,” “obesity,” “hip fracture,” and “mortality.” The fifth cluster is shown in purple and reflects content related to both systemic evaluation and blood loss, with key words including “hip arthroplasty,” “systematic review,” “knee arthroplasty,” “tranexamic acid,” “transfusion,” and “blood loss.” The sixth cluster is shown in cyan and relates to complications and cost, with key words including “complication,” “TKA,” “risk factor,” “readmission,” “length of stay,” and “cost.” Early themes include antibiotic prophylaxis, epidemiology, and sonication. In recent years, popular themes have included instability, survivorship, and CRP (Fig. 8B). We further utilized CiteSpace to analyze the burst words and the top 25 are presented in Fig. 8C. The word with the highest burst intensity was “orthopedic surgery,” which had a burst period from 2012 to 2016. The word with the longest burst period was “resistant staphylococcus aureus,” which had a burst period from 2012 to 2017. Finally, we also list the affiliation between authors and keywords in the field of PJI research after primary THA in Fig. 8D.

## Discussion

### Meta-analysis

Our research has revealed a significant disparity between the incidence estimates of PJI derived from databases and those based on clinical assessments. Specifically, while the former stands at a modest 1.05%, the latter indicates a higher incidence of 1.74%. A variety of factors may contribute to this discrepancy, but among them, we posit that differences in management practices across databases may be particularly salient.

Problems in database studies: (1) Of the database-based studies we included, 5 [12, 14, 21, 22, 26] had data from databases that recorded only revision patients. Considering that PJI can be treated not only by revision but also by long-term antibiotic therapy, debridement, joint fusion, and amputation treatment methods, we believe this is one of the reasons for the underestimation of the incidence of PJI in the database studies. (2) Although the included database-based studies were published after 2012, the patients in them had hip arthroplasty spanning a long period of time (1995–2018) and there were differences in the diagnostic methods used. For example, in the diagnostic code, Weinstein [32] in the United States reported a Positive predictive value of 53.8% (43/80) for the diagnosis of PJI by ICD-9, compared with 60% (48/80) for ICD-10. (3) Procedural issues are also an important reason for the low reported rate of readmission for PJI after initial THA. Lindgren [33] in Sweden found a 67% reporting rate of PJI in the Swedish Hip Arthroplasty Register. Zhu [34] reported New Zealand Joint Registry data underestimates the rate of PJI and the readmission reporting rate was missing by one-third. In particular, in some studies involving earlier records, it is possible that acute infections occurring during postoperative hospitalization for THA were described as in-hospital events, resulting in omissions [35]. Such man-made disparities, if not addressed, may have serious implications for public health interventions and clinical decision-making. We therefore advocate for further investigation into the underlying causes of the differences in PJI incidence estimates, and the development of standardized protocols for data collection and analysis across different platforms. By doing so, we can minimize the impact of artificial variability and enable more accurate and reliable assessments of PJI incidence and its associated risk factors.

This study found that the mean age of patients who developed PJI in the database-based study was between 60 and 70 years. the incidence was slightly higher in patients > 70 years (1.90%) than in patients < 70 years (1.68%). The majority of patients requiring arthroplasty are > 50 years of age [36, 37]. Patients in this age group tend to have underlying diseases and a weaker immune system than younger patients, and the probability of infection may be higher in older patients than in younger patients. However, a META analysis suggests that age may also be one of the protective factors [38].

We also identified a lower incidence of PJI in women (1.58%) than in men(2.07%) in database-based studies, but a lower incidence of PJI in men(0.38%) than in women(0.94%) in clinic-based studies. Because the number of men and women was reported in full in fewer included studies, the risk of bias is high and should be considered with caution.

Most patients with PJI occurred within 90 days after THA (1.43%). PJI after more than 90 days is defined as chronic infection [39]. Our study found that the incidence of PJI over 90 days to 1 year was 0.49%. Muscatelli [19] reports on data centers in multiple countries monitoring PJI for 90 days. This leaves data missing for patients with PJI beyond 90 days. To fully capture these infections and track trends, while identifying opportunities for quality improvement, we suggest that infection surveillance should be extended to 1 year [40, 41].

In our analysis of the incidence of PJI across continents, we found high heterogeneity in the incidence of PJI in Europe based on database studies. This includes the aforementioned database discrepancies, inconsistent diagnostic codes, and procedural issues. The problem of data omission has been a nuisance, and numerous studies have complained about underestimation of the incidence of PJI obtained from database analysis [35, 42–44]. The validity of the joint replacement database depends on the coverage and completeness of the data entered into the database. Coverage is important, but if completeness is low for some or all operating units, these numbers may be misleading. This could lead to an underestimation of the true total incidence of reoperations and result in incorrect relative incidence between individual reporting units. Therefore, it is important to assess coverage and completeness in order to fairly interpret the results. Interestingly, we found that the majority of the included studies were from developed countries and only 3 studies [17, 18, 24] were from non-developed countries. All of non-developed countries reported a high incidence of PJI. There are fewer studies on the correlation between the regional economy and the incidence of PJI, and further confirmation is needed.

### Bibliometric analysis

The development of diagnostic criteria for PJI in 2011 by the American Musculoskeletal Infection Association has led to an increasing number of articles on PJI after THA. Globally, there has been a rising trend in PJI-related literature since 1998, as reported by previous studies [45]. Notably, a rapid increase in the growth of literature was observed at three specific time points: 2013, 2015, and 2018. This trend may be attributed to significant events in the field of PJI research, such as the 1st [39] and 2nd [3] International Consensus Conferences on Artificial Joint Infection held in 2013 and 2018, respectively. These conferences likely served as a platform for knowledge exchange and collaborative efforts among researchers, leading to an increased interest and output of PJI-related literature.

In this bibliometric analysis, the most relevant articles were found to have corresponding authors from multiple countries, including the United States, China, Germany, and the United Kingdom. This trend is commonly observed in other bibliometric studies and can be attributed to the contribution of national economic power to medical research [46]. Additionally, countries with higher rates of joint replacement surgeries are more likely to have a higher number of PJI cases, leading to increased research interest and output in these areas. For instance, in 2017, the number of hip replacements (including primary replacement and revision) per 100,000 people reached 194 in the United States, 281 in Germany, and 182 in the United Kingdom. In China, although the number was only 39.65 in 2018, this may be attributed to the country's larger population base. In fact, the number of hip replacements performed in China in 2018 (39.65) was much higher than that in Germany during the same year (22.3). These figures highlight the differences in joint replacement rates among countries, which can influence the incidence of PJI and subsequent research activity.

There is a significant amount of collaboration between countries in research related to PJI after hip arthroplasty. One notable collaboration is between the United States and China, which can be attributed to the large number of patients and specialists in both countries, as well as their strong research capacity and infrastructure. The academic exchange between the two countries is also frequent, and there are many joint journals and conferences. Comparing different types of repair procedures, such as single or dual stage, is also a common interest between the United States and China. Another collaboration worth mentioning is between the United States and Israel, which is likely due to Israel's high level of innovation and international influence in the biomedical field. Israel has many scientific institutions and talented researchers with close ties to the United States. The strong geopolitical and cultural ties between the United States and Canada may also explain their collaboration in PJI research. Both countries have similar healthcare systems and standards, making academic exchanges between them easier. There are also many cross-border journals and conferences. The collaboration between the United States and Germany is likely due to Germany's extensive experience in hip arthroplasty, excellent technology and quality, and status as one of the largest investors in research in Europe. Germany also has a good relationship with the United States, further facilitating collaboration in PJI research.

It is noteworthy that almost all of the top research institutions in this field are located in the United States, which can be partly explained by factors such as the country's strong economy and high volume of joint replacement surgeries. For example, the Mayo Clinic, which is one of the leading medical institutions in the world, was named "World's Best Hospital" by Newsweek [47]. This recognition reflects the institution's expertise and reputation in the field of orthopedics, including research on PJI after THA.

The top ten most published journals in the field of PJI after THA are predominantly based in Europe and North America, reflecting the concentration of research activity in these regions. These journals have impact factors that range from 0.8 to 5.2 in the last two years, indicating the quality and influence of their published research. Examples of these top journals include Clinical Orthopaedics and Related Research, The Journal of Arthroplasty, and The Bone & Joint Journal. These journals have a long history of publishing cutting-edge research in the field of orthopedics and related specialties, and their high impact factors reflect the quality of their published work.

Javad Parvizi, a researcher from Thomas Jefferson University in the USA, holds the record for the highest number of publications in the field of PJI for both knee and hip replacements [45]. His team has established numerous collaborations with other countries in the field of PJI research, including Israel [3], Germany [48], and China [49].

“Diagnosis and Management of Artificial Joint Infections: Clinical Practice Guidelines” is the most cited literature on the subject. This article focuses on the diagnosis and treatment of PJI. It covers a broad range of topics including the definition of infection, underlying causes, clinical manifestations, diagnostic techniques, and treatment options. The guidelines provide comprehensive guidance to healthcare providers for effectively managing PJIs, with a strong emphasis on accurate diagnosis through the use of blood markers and tissue cultures. Treatment modalities mentioned in the guidelines include surgical debridement, antibiotic therapy, and prosthetic joint removal.

VOSviewer is a powerful tool for visually analyzing author keywords and generating knowledge maps that illustrate the knowledge structure and evolution of a field or topic. Based on recent research, the major themes in PJI after THA can be summarized as follows: (1) The use of antibiotic bone cement in preventing and treating PJI, including the selection of bone cement and antibiotics, the nature of the compound after it is formed, and its clinical efficacy. (2) Diagnostic studies of PJI, including the sensitivity, specificity, accuracy, negative predictive value, and false-positive rate of various diagnostic tools. (3) Diagnostic criteria for PJI, including the validity and limitations of the MSIS criteria and other possible diagnostic methods. (4) Bacterial spectrum and drug resistance of PJI, including common causative organisms, multi-drug resistant organisms, and methicillin-resistant Staphylococcus aureus. In recent years, the detection of drug-resistant bacteria, including MRSA, after the initial joint replacement or even multiple revisions, remains a common occurrence [50, 51]. (5) Treatment strategies for PJI, including stage I or II revision, DAIR, antibiotic regimens, and prognostic evaluation.

## Limitations

In meta-analysis, the present study has certain limitations that should be acknowledged. Firstly, the high heterogeneity of database-based studies prevented us from obtaining homogenous estimates of PJI incidence, despite acknowledging the differences in methodology and outcomes between database-based and clinical research. Secondly, due to the limited number of studies in certain subgroups, our analysis may have gaps in search coverage, although our funnel plot analysis indicates minimal impact on the results. Thirdly, one [16] of the clinical-based studies lacked the reporting of their diagnostic criteria. Lastly, the overall quality of the included articles is not optimal, and caution is advised when interpreting the results.

In bibliometric analysis, although the WOS is widely regarded as the most reliable database, it is important to note that some articles may still be missed. This can occur due to a variety of reasons, such as incomplete coverage or errors in indexing. Additionally, the majority of articles included in WOS are published in English, which may introduce selection bias in terms of language of publication, as not all research is published in English.

## Conclusion

The combined incidence of PJI was lower in database-based studies than in clinic-based studies. The incidence of PJI in database-based studies is underestimated. Our findings highlight the significance of carefully considering the choice of data source when estimating PJI incidence. The variability in incidence estimates derived from different sources underscores the importance of establishing standardized diagnostic criteria and surveillance methods to facilitate more reliable and accurate assessment of PJI rates. In the same time, the bibliometric study on PJI following THA has shown that the number of articles in this field is increasing rapidly. The United States is the country with the highest number of publications in this area, and the Mayo Clinic in the US is the institution that has made the greatest scientific impact in PJI research. The most influential journal in this field is "Diagnosis and Management of Artificial Joint Infections: Clinical Practice Guidelines," and there is a noticeable shift from diagnostic to prognostic research in this field. The implications of these findings for clinical practice and policy warrant further investigation, as they could potentially have an impact on resource allocation, patient outcomes, and public health initiatives.

### Supplementary Information

**Additional file 1**. **Fig. 1**: Forest plot of incidence of PJI by Search criteria in the database based studies. **Fig. 2**: Forest plot of incidence of PJI by age in the database based studies. **Fig. 3**: Forest plot of incidence of PJI by sex in the database based studies. **Fig. 4**: Forest plot of incidence of PJI by time to post-THA infection in the database based studies. **Fig. 5**: Scatterplot of the incidence of PJI by publication time in the database-based studies. **Fig. 6**: Scatterplot of the incidence of PJI by publication time in the clinic-based studies. **Fig. 7**: Forest plot of incidence of PJI by continent in the database-based studies. **Fig. 8**: Forest plot of incidence of PJI by continent in the clinic-based studies. **Fig. 9**: Publication bias of the database-based studies incidence studies of PJI. **Fig. 10**: Publication bias of the database-based studies incidence studies of PJI. **Fig. 11**: Publication bias of the database-based studies incidence studies of PJI (Egger test). **Fig. 12**: Publication bias of the clinic-based studies incidence studies of PJI. **Fig. 13**: Publication bias of the clinic-based studies incidence studies of PJI (Egger test). **Fig. 14**: Sensitivity Analysis of the database-based studies incidence studies of PJI. **Fig. 15**: Sensitivity Analysis of the clinic-based studies incidence studies of PJI. **Fig. 16**: The annual number of publications in the most influential Journal. The horizontal coordinate is the year and the vertical coordinate is the cumulative number of articles issued. **Table 1**: Search Strategy. **Table 2**: Quality assessment. **Table 3**: Dabtabase-based studies correlations analysis. **Table 4**: Clinic-based studies correlation analysis. **Table 5**: Meta-regression of the incidence of PJI in database-based studies. **Table 6**: The top 10 most productive research institutions. **Table 7**: the top 10 journals with the highest number of publications. **Table 8**: The top 10 authors with the highest number of publications. **Table 9**: the 50 most frequently occurring author keywords.

## Data Availability

The data used for this meta-analysis and bibliometrics are publicly available. The search strategies, screening results, quality assessment, and meta-analysis results can be downloaded from the journal website or Pubmed, Web of Science and Embase.
