# Supplementary material for: Incidence of periprosthetic joint infection after primary total hip arthroplasty is underestimated: a synthesis of meta-analysis and bibliometric analysis
Source: J Orthop Surg Res. 2023 Aug 21;18:610. doi: 10.1186/s13018-023-04060-5 (PMC10440885; doi:10.1186/s13018-023-04060-5)
Supplement: Supplementary file 1 — Additional file 1. Fig. 1: Forest plot of incidence of PJI by Search criteria in the database based studies. Fig. 2: Forest plot of incidence of PJI by age in the database based studies. Fig. 3: Forest plot of incidence of PJI by sex in the database based studies. Fig. 4: Forest plot of incidence of PJI by time to post-THA infection in the database based studies. Fig. 5: Scatterplot of the incidence of PJI by publication time in the database-based studies. Fig. 6: Scatterplot of the incidence of PJI by publication time in the clinic-based studies. Fig. 7: Forest plot of incidence of PJI by continent in the database-based studies. Fig. 8: Forest plot of incidence of PJI by continent in the clinic-based studies. Fig. 9: Publication bias of the database-based studies incidence studies of PJI. Fig. 10: Publication bias of the database-based studies incidence studies of PJI. Fig. 11: Publication bias of the database-based studies incidence studies of PJI (Egger test). Fig. 12: Publication bias of the clinic-based studies incidence studies of PJI. Fig. 13: Publication bias of the clinic-based studies incidence studies of PJI (Egger test). Fig. 14: Sensitivity Analysis of the database-based studies incidence studies of PJI. Fig. 15: Sensitivity Analysis of the clinic-based studies incidence studies of PJI. Fig. 16: The annual number of publications in the most influential Journal. The horizontal coordinate is the year and the vertical coordinate is the cumulative number of articles issued. Table 1: Search Strategy. Table 2: Quality assessment. Table 3: Dabtabase-based studies correlations analysis. Table 4: Clinic-based studies correlation analysis. Table 5: Meta-regression of the incidence of PJI in database-based studies. Table 6: The top 10 most productive research institutions. Table 7: the top 10 journals with the highest number of publications. Table 8: The top 10 authors with the highest number of publications. Table 9: the 50 most frequently occurring author keywor [file 13018_2023_4060_MOESM1_ESM.docx]

Supplementary Material

Incidence of periprosthetic joint infection after primary total hip arthroplasty is underestimated

**A synthesis of meta-analysis and bibliometric analysis**

## Supplementary Figures
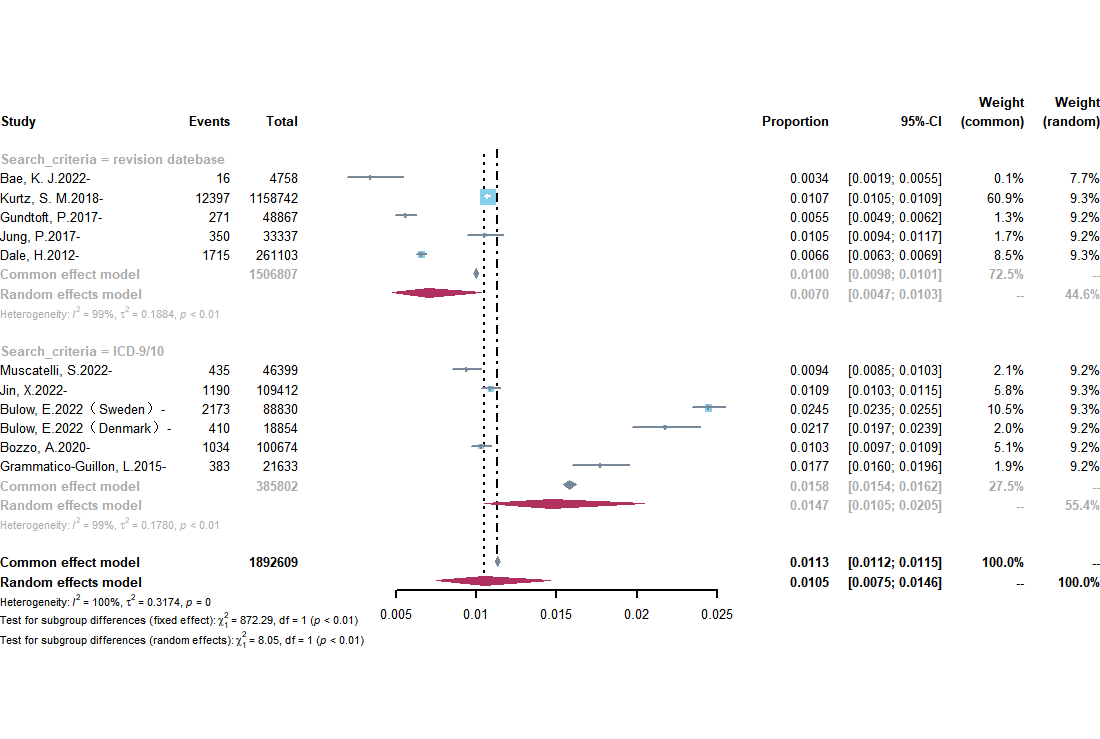
Supplementary Figure 1. Forest plot of incidence of PJI by Search criteria in the database based studies

.
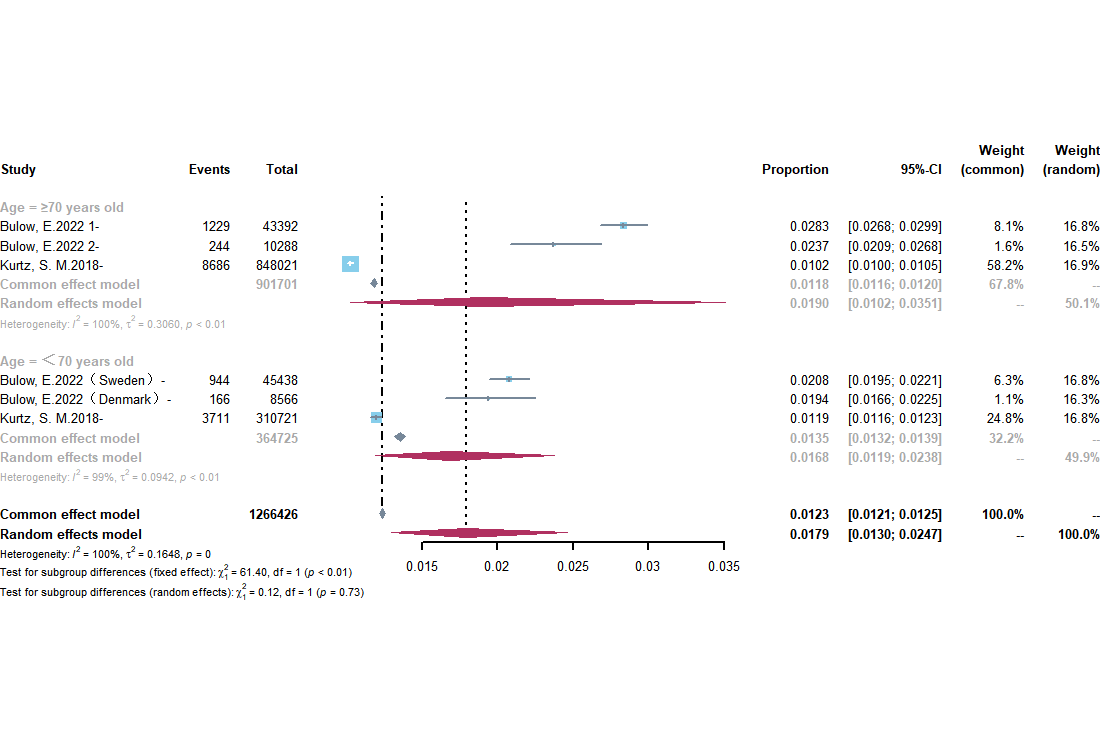


**Supplementary Figure 2 Forest plot of incidence of PJI by age in the database based studies**

**
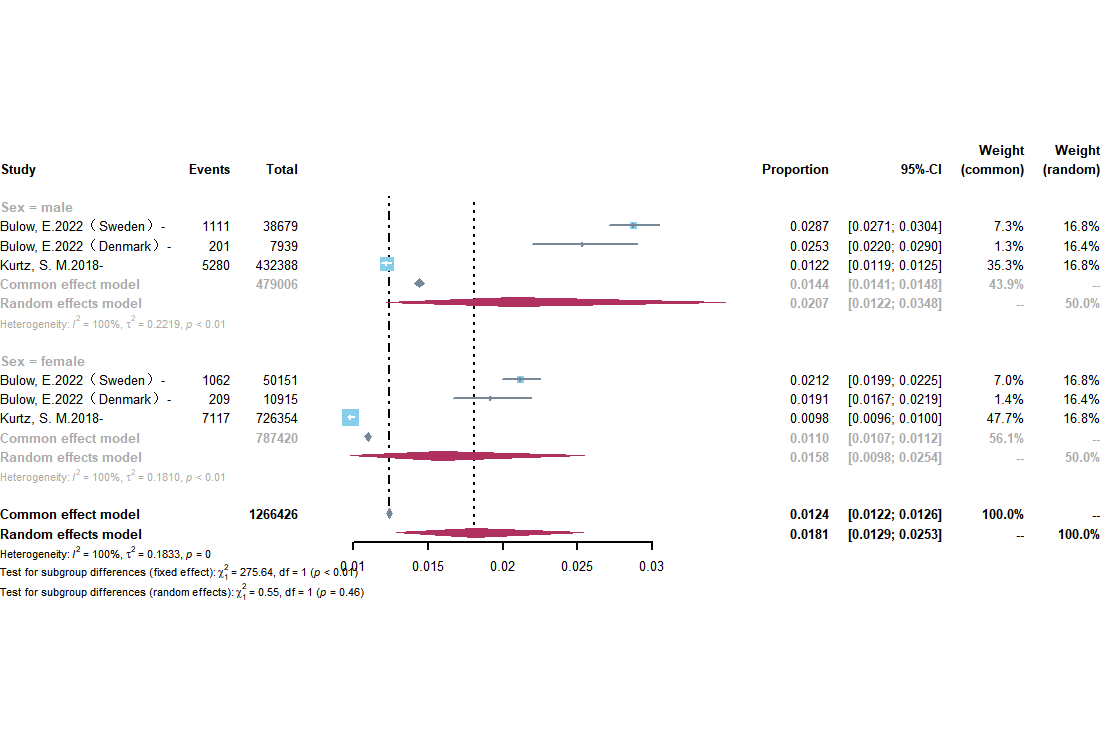
**

**Supplementary Figure 3 Forest plot of incidence of PJI by sex in the database based studies**

**
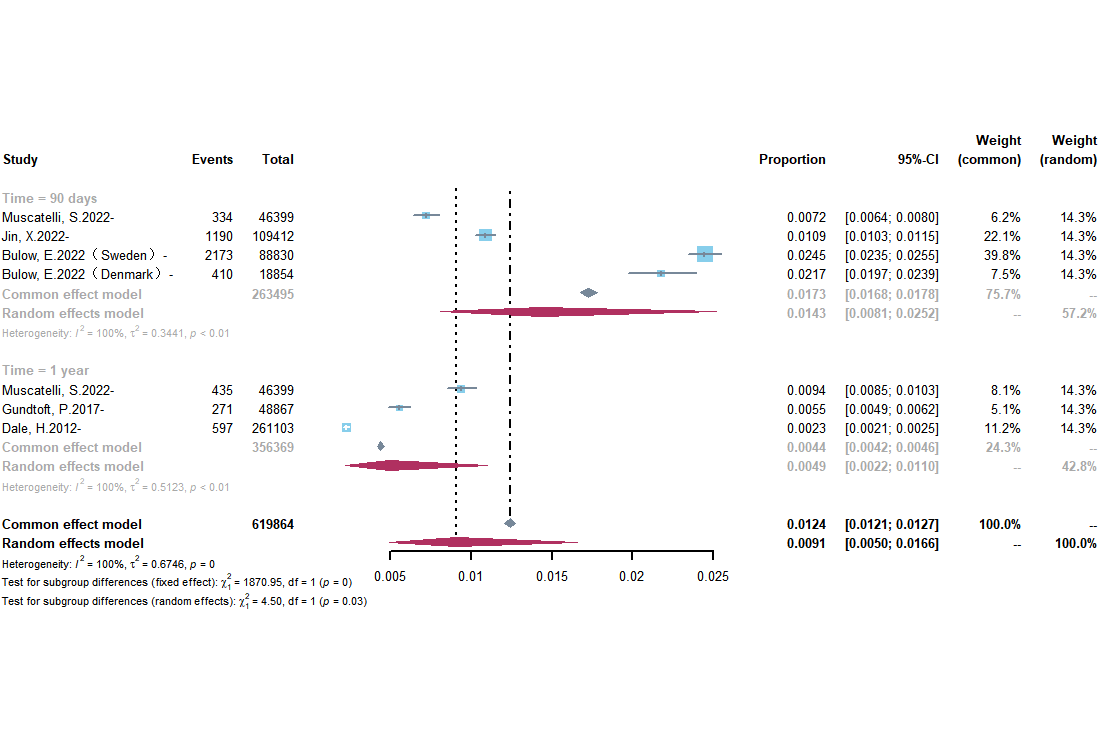
**

**Supplementary Figure 4 Forest plot of incidence of PJI by time to post-THA infection in the database based studies**

**
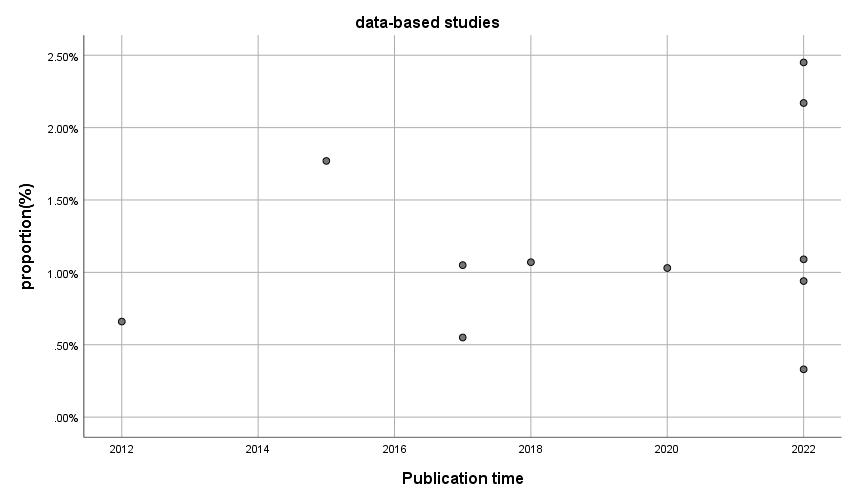
**

**Supplementary Figure 5 Scatterplot of the incidence of PJI by publication time in the database-based studies**


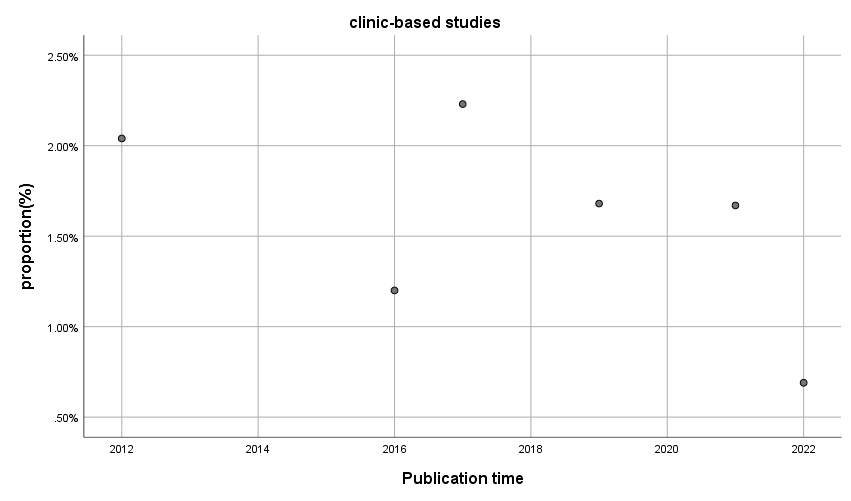


**Supplementary Figure 6 Scatterplot of the incidence of PJI by publication time in the clinic-based studies**


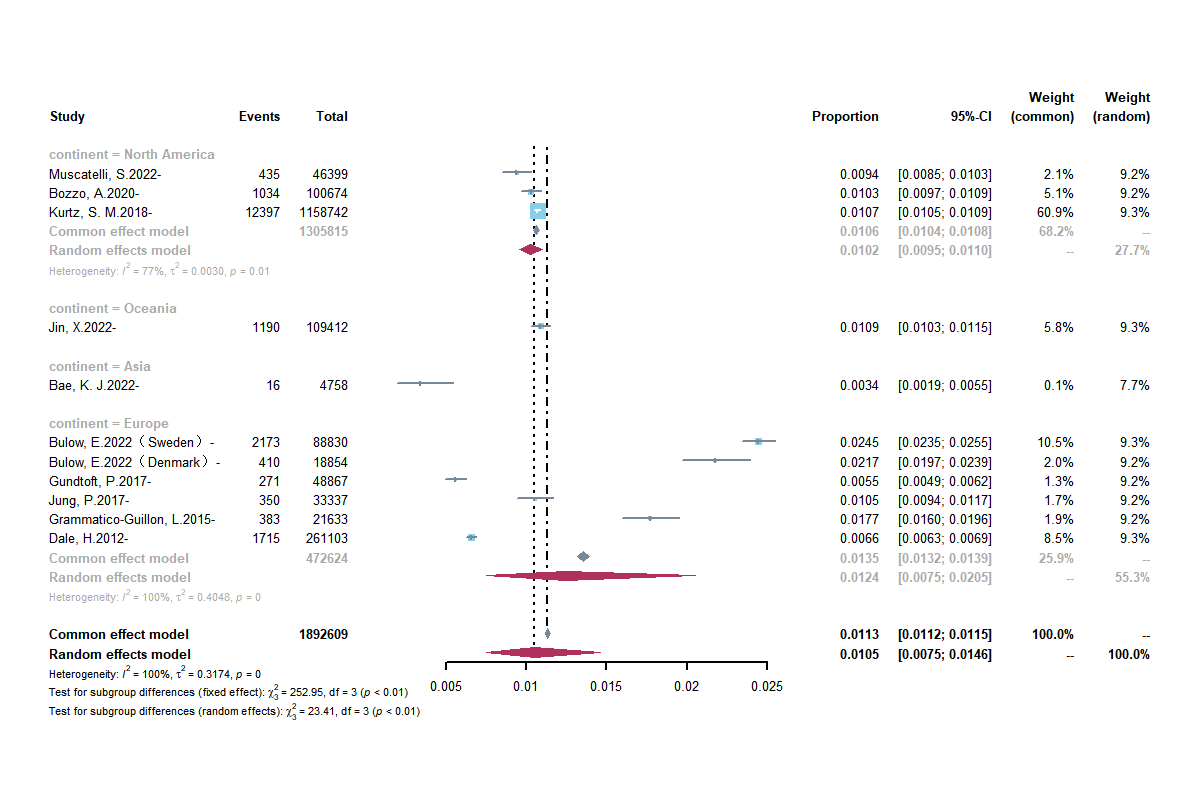


**Supplementary Figure 7 Forest plot of incidence of PJI by continent in the database-based studies**


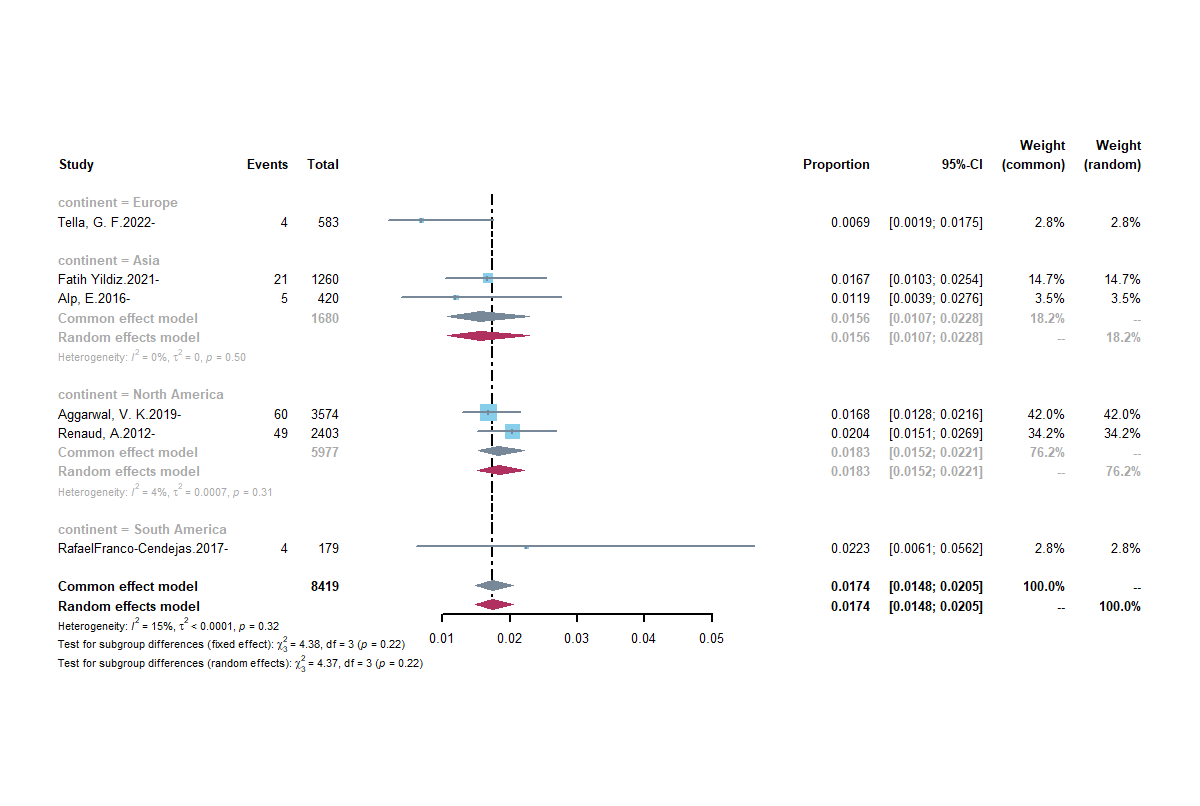


**Supplementary Figure 8 Forest plot of incidence of PJI by continent in the clinic-based studies**


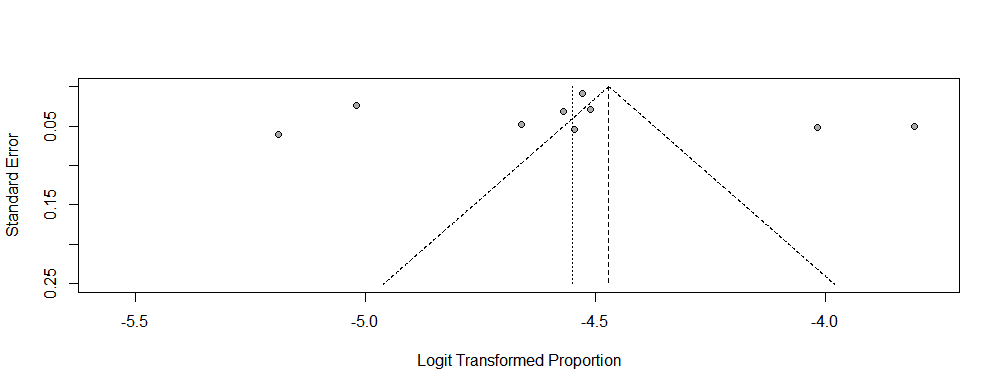


**Supplementary Figure 9 Publication bias of the database-based studies incidence studies of PJI**

**
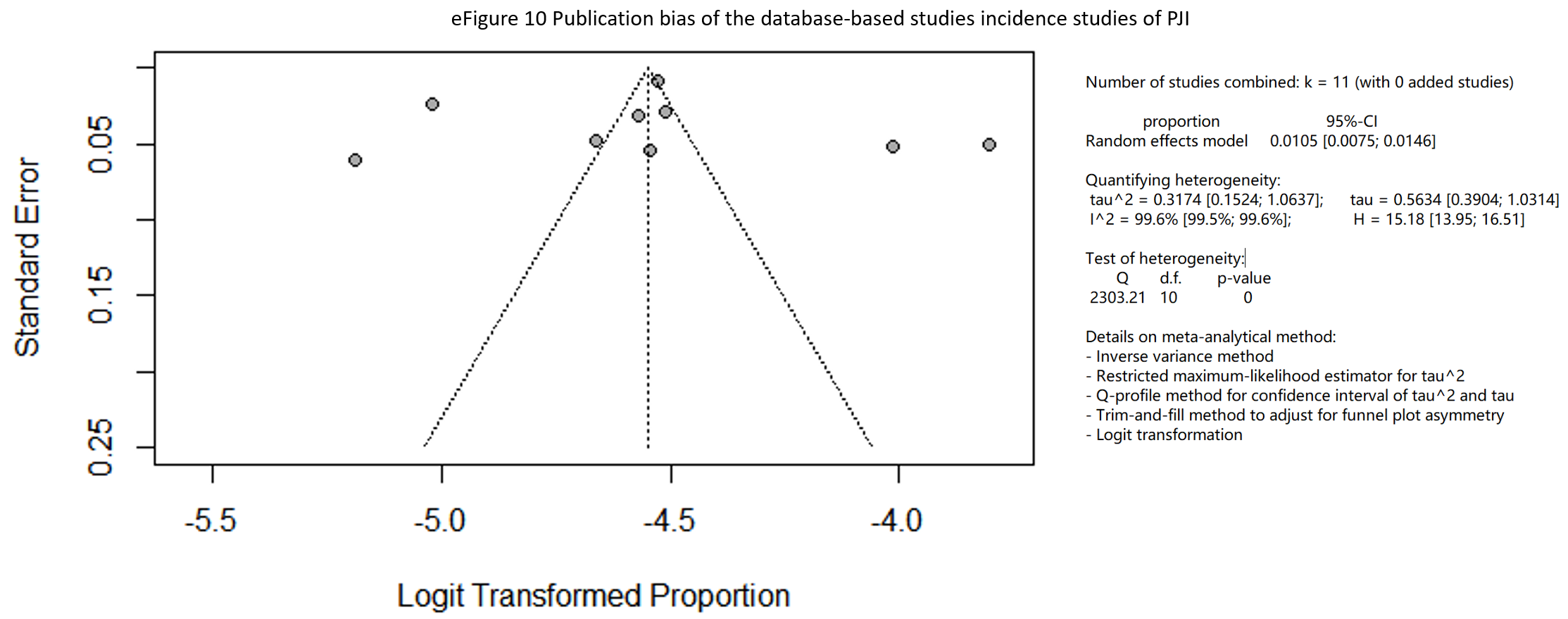
**

**Supplementary Figure 10 Publication bias of the database-based studies incidence studies of PJI**

**
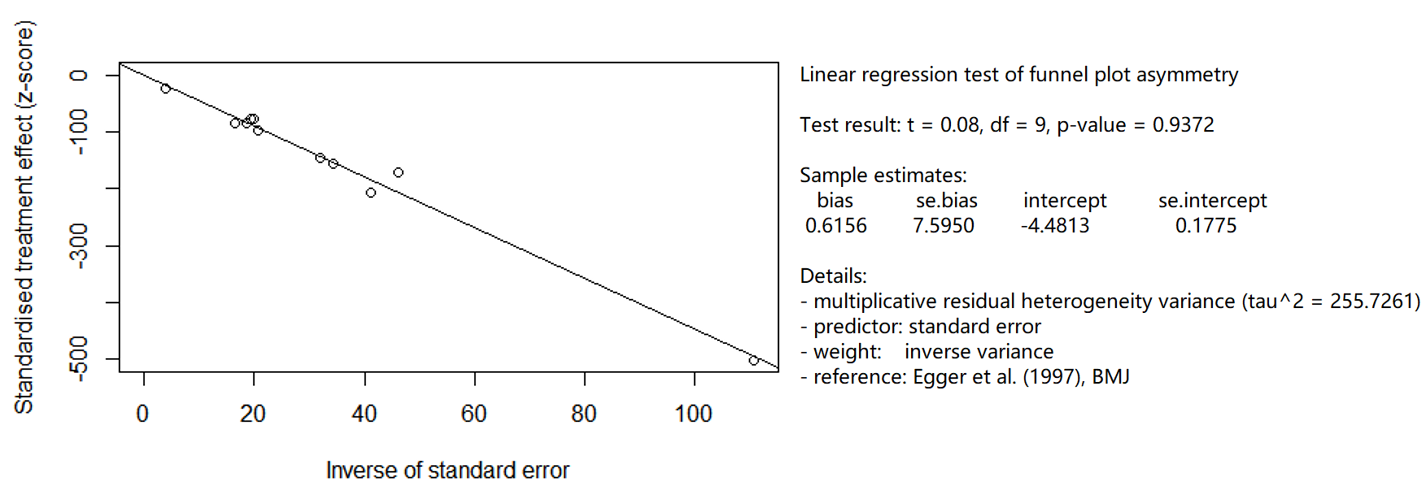
**

**Supplementary Figure 11 Publication bias of the database-based studies incidence studies of PJI (Egger test)**

**
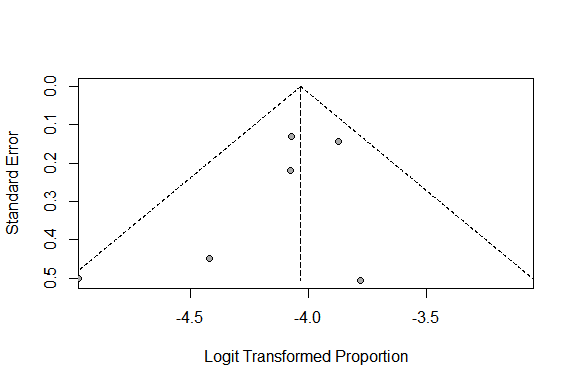
**

**Supplementary Figure 12 Publication bias of the clinic-based studies incidence studies of PJI**

**
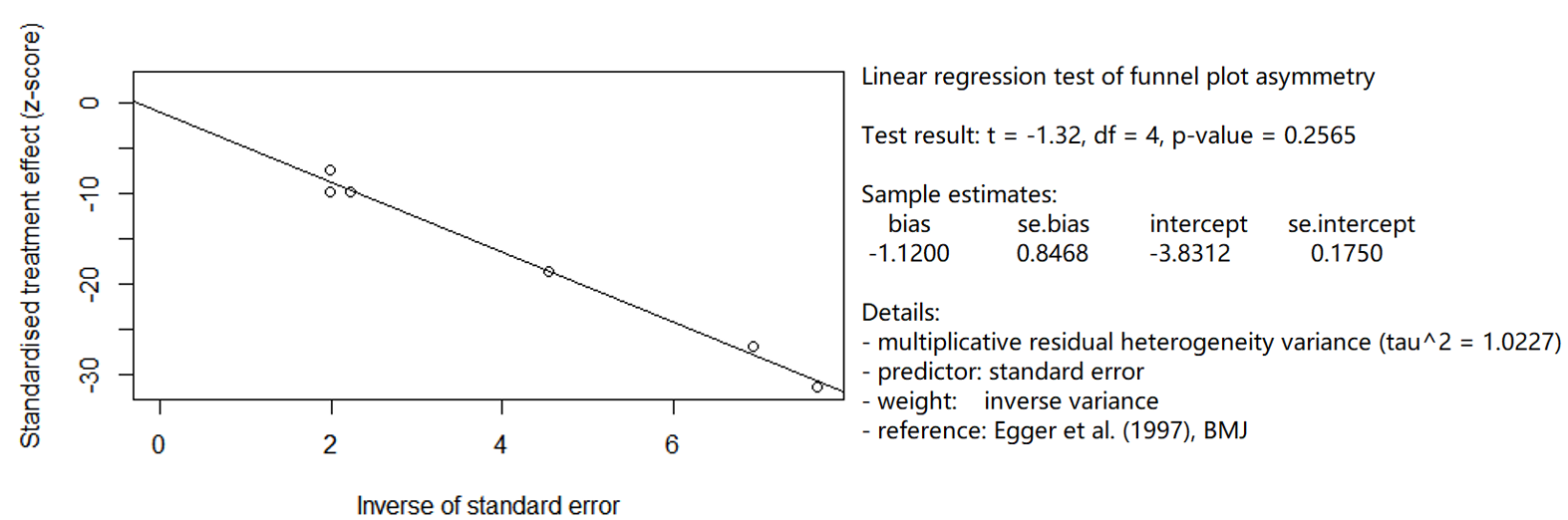
**

**Supplementary Figure 13 Publication bias of the clinic-based studies incidence studies of PJI (Egger test)**

**
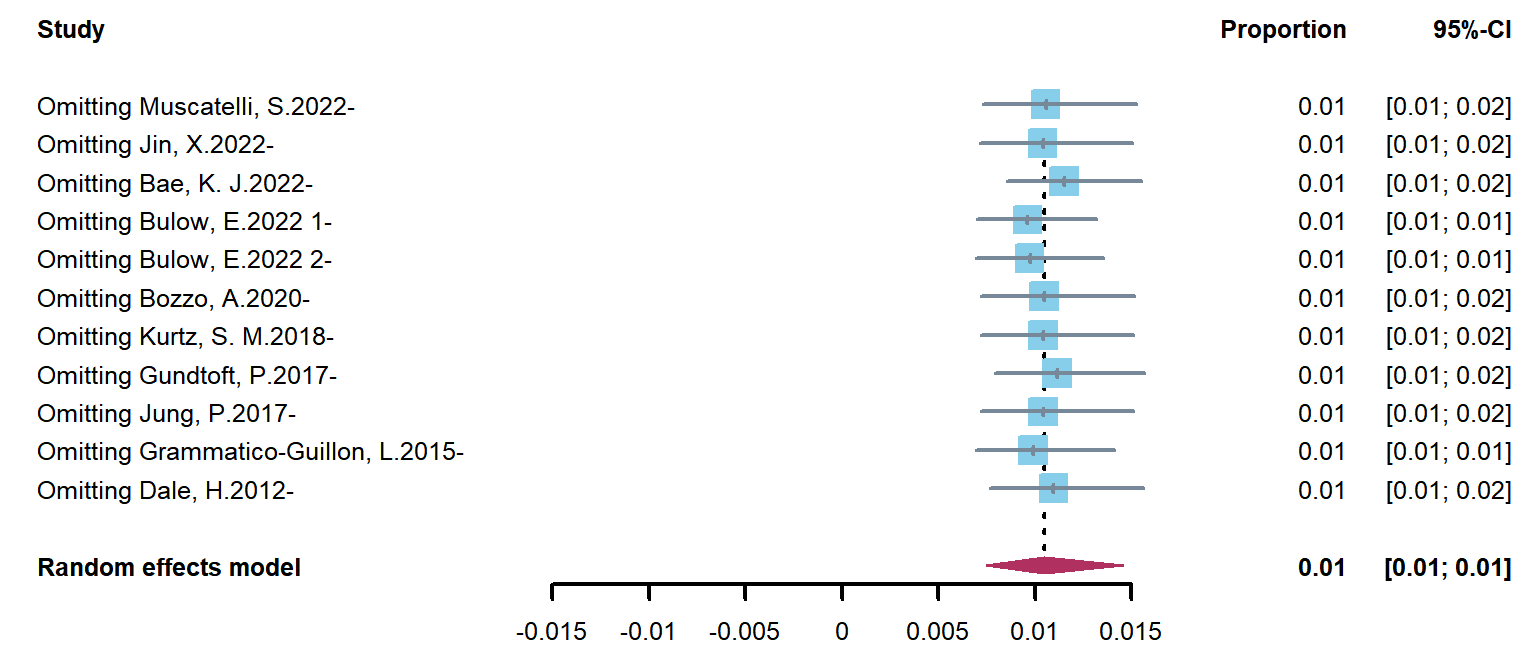
**

**Supplementary Figure 14 Sensitivity Analysis of the database-based studies incidence studies of PJI**

**
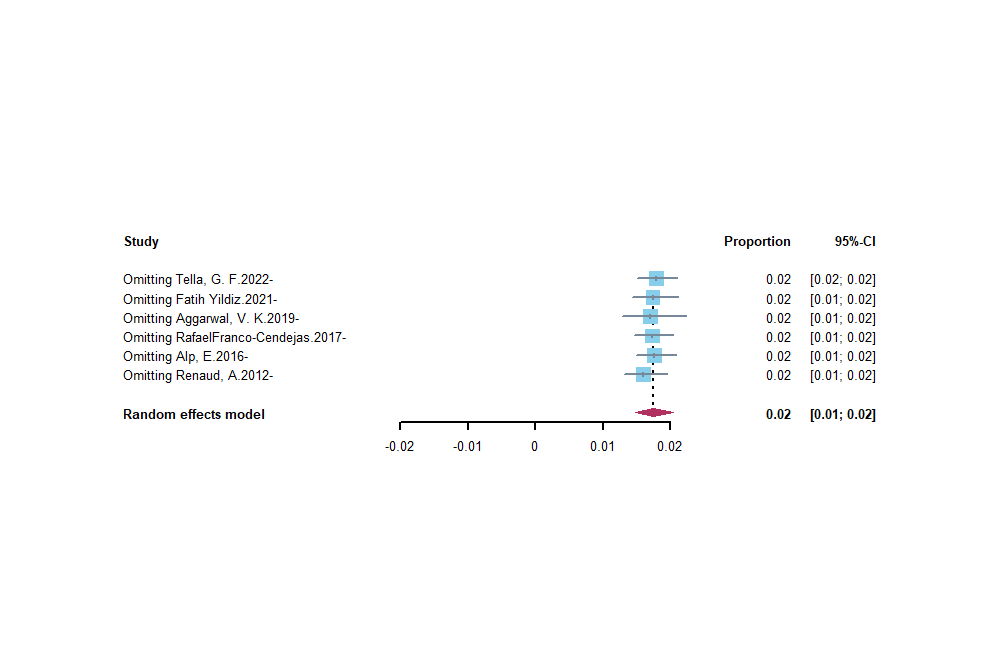
**

**Supplementary Figure 15 Sensitivity Analysis of the clinic-based studies incidence studies of PJI**

**Supplementary Figure 16 The annual number of publications in the most influential**

**Journal. The horizontal coordinate is the year and the vertical coordinate is the cumulative number of articles issued.**

## Supplementary Table


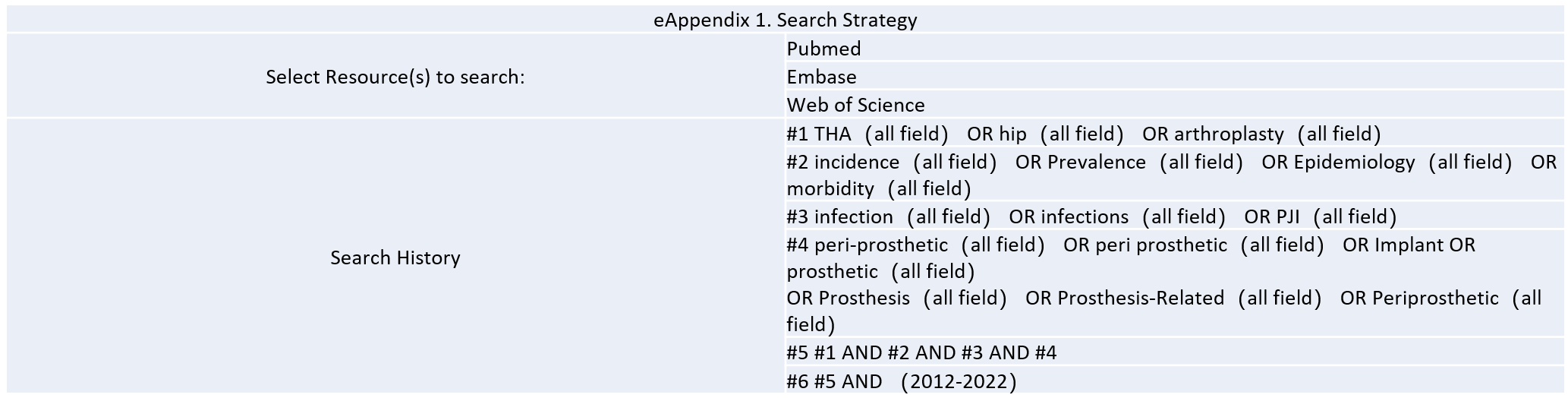


**Supplementary Table 1 Search Strategy**

| **Author,year** | **Q1** | **Q2** | **Q3** | **Q4** | **Q5** | **Q6** | **Q7** | **Q8** | **Q9** | **Total(%)** | **Risk of Bias** |
| --- | --- | --- | --- | --- | --- | --- | --- | --- | --- | --- | --- |
| **Muscatelli, S.2022** | **Y** | **U** | **Y** | **U** | **Y** | **Y** | **U** | **Y** | **Y** | **66.67** | **MODERATE** |
| **Tella, G. F.2022** | **U** | **U** | **U** | **U** | **U** | **Y** | **Y** | **Y** | **Y** | **44.44** | **HIGH** |
| **Bae, K. J.2022** | **U** | **Y** | **Y** | **U** | **U** | **Y** | **U** | **Y** | **U** | **44.44** | **HIGH** |
| **Bulow, E.2022** | **Y** | **Y** | **Y** | **U** | **Y** | **Y** | **Y** | **Y** | **Y** | **88.89** | **LOW** |
| **Jin, X.2022** | **Y** | **U** | **Y** | **U** | **Y** | **N** | **U** | **Y** | **U** | **44.44** | **HIGH** |
| **Fatih Yildiz.2021** | **U** | **Y** | **U** | **U** | **U** | **Y** | **U** | **Y** | **U** | **33.33** | **HIGH** |
| **Bozzo, A.2020** | **Y** | **Y** | **Y** | **U** | **Y** | **Y** | **U** | **Y** | **N** | **66.67** | **MODERATE** |
| **Aggarwal, V. K.2019** | **U** | **Y** | **U** |  | **Y** | **U** | **Y** | **Y** | **Y** | **66.67** | **MODERATE** |
| **Kurtz, S. M.2018** | **Y** | **Y** | **Y** | **Y** | **Y** | **Y** | **U** | **Y** | **U** | **77.78** | **LOW** |
| **Gundtoft, P. H.2017** | **Y** | **U** | **Y** | **Y** | **Y** | **N** | **U** | **Y** | **U** | **55.56** | **MODERATE** |
| **Franco-Cendejas, R.2017** | **U** | **Y** | **U** | **Y** | **U** | **Y** | **Y** | **Y** | **N** | **55.56** | **MODERATE** |
| **Jung, P.2017** | **Y** | **Y** | **Y** | **U** | **Y** | **N** | **U** | **Y** | **Y** | **66.67** | **MODERATE** |
| **Alp, E.2016** | **U** | **U** | **U** | **Y** | **U** | **Y** | **Y** | **Y** | **U** | **44.44** | **HIGH** |
| **Grammatico-Guillon, L.2015** | **U** | **Y** | **Y** | **U** | **U** | **Y** | **N** | **Y** | **U** | **44.44** | **HIGH** |
| **Dale, H.2012** | **Y** | **Y** | **Y** | **U** | **Y** | **N** | **Y** | **Y** | **U** | **66.67** | **MODERATE** |
| **Renaud, A.2012** | **U** | **Y** | **U** | **Y** | **U** | **Y** | **Y** | **Y** | **U** | **55.56** | **MODERATE** |
| **Legend:Y=Yes;N=No;U=Unclear;NA=Not applicable** | | | | | | | | | | | |
| **Risk of bias was categorized as high when the study reaches up to 49% score "yes" ,moderate wthen the study reached 50% to 69%** | | | | | | | | | | | |
| **score "yes" ,and low when the study reached more than 70% score "yes".** | | | | | | | | | | | |

**Supplementary Table 2 Quality assessment**

**
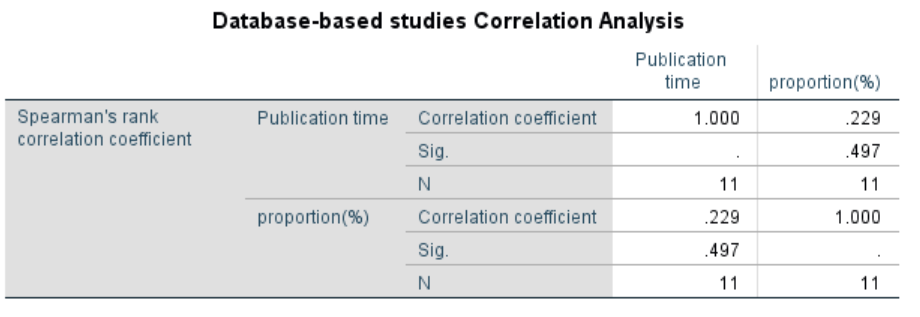
**

**Supplementary Table 3 Dabtabase-based studies correlations Analysis**

**
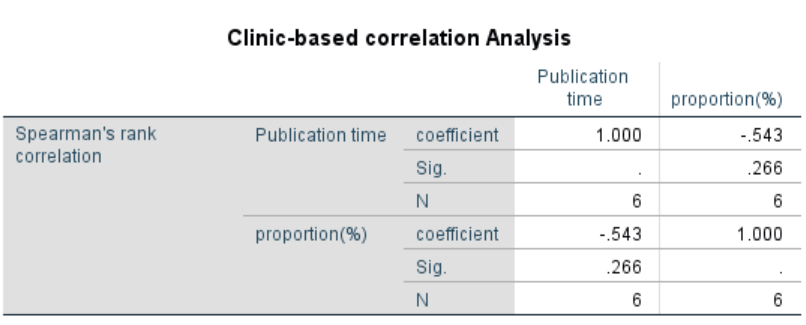
**

**Supplementary Table 4 Clinic-based studies correlation Analysis**


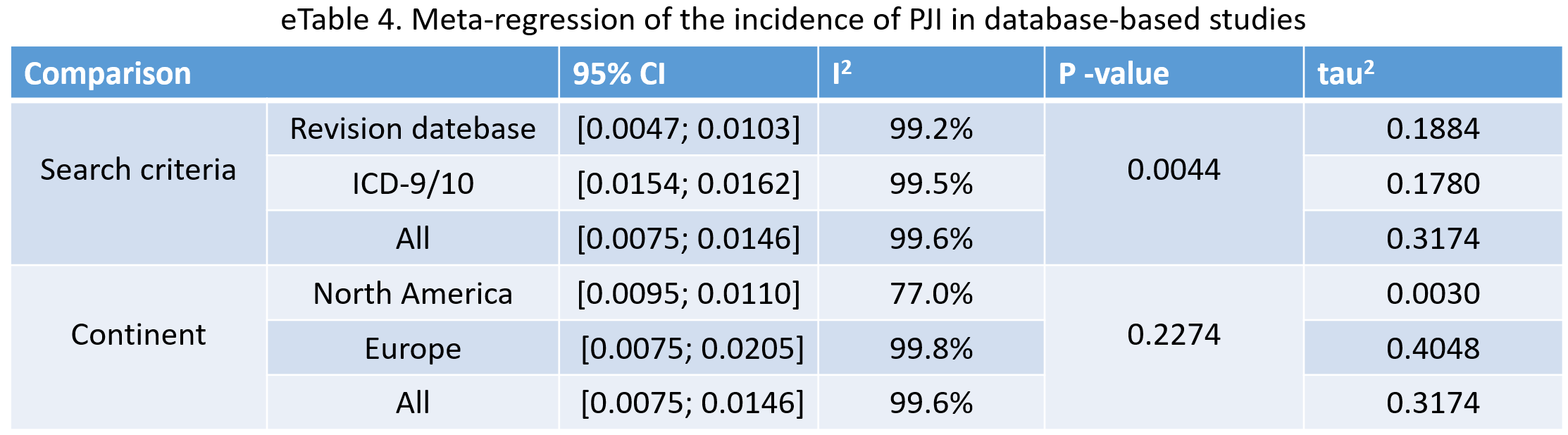


**Supplementary Table 5 Meta-regression of the incidence of PJI in database-based studies**

| The top 10 most productive research institutions | | |
| --- | --- | --- |
| Institution | Articles | Country |
| Mayo Clinic | 200 | US |
| Thomas Jefferson University | 161 | US |
| Hospital for Special Surgery | 132 | US |
| Rush University | 103 | US |
| Harvard Medical School | 78 | US |
| Cleveland Clinic | 75 | US |
| Duke University | 73 | US |
| Sichuan University | 73 | CN |
| University of Toronto | 62 | CA |
| University of Pennsylvania | 61 | US |

**Supplementary Table 6 The top 10 most productive research institutions**

| the top 10 journals with the highest number of publications | | | | | |
| --- | --- | --- | --- | --- | --- |
| Journal Name | articles | Country of Publication | 2022 Impact Factor | 2023 Impact Factor | JCR Category |
| *Journal of Arthroplasty* | 914 | USA | 3.85 | 4.435 | Q1 (Surgery) Q2 (Orthopedics and Sports Medicine) |
| *Bone & Joint Journal* | 184 | UK | 4.306 | NA | Q1 (Surgery) Q1 (Orthopedics and Sports Medicine) |
| *Hip International* | 180 | UK | 1.802 | NA | Q3 (Surgery) Q3 (Orthopedics and Sports Medicine) |
| *International Orthopaedics* | 151 | Germany | 2.724 | NA | Q2 (Surgery) Q2 (Orthopedics and Sports Medicine) |
| *Journal of Bone and Joint Surgery-American Volume* | 147 | USA | 5.163 | NA | Q1 (Surgery) Q1 (Orthopedics and Sports Medicine) |
| *Archives of Orthopaedic and Trauma Surgery* | 115 | Germany | 1.977 | NA | Q3 (Surgery) Q3 (Orthopedics and Sports Medicine) |
| *Clinical Orthopaedics and Related Research* | 115 | USA | 4.154 | NA | Q1 (Surgery) Q1 (Orthopedics and Sports Medicine) |
| *Orthopedics* | 90 | USA | 0.862 | NA | Q4 (Surgery) Q4 (Orthopedics and Sports Medicine) |
| *BMC Musculoskeletal Disorders* | 82 | UK | 2.239 | NA | Q2（Orthopedics and Sports Medicine） |
| ACTA Orthopaedica | 71 | Sweden | 3.057 | NA | Q1（Surgery） Q1（Orthopedics and Sports Medicine） |

**Supplementary Table 7 the top 10 journals with the highest number of publications**

| The top 10 authors with the highest number of publications | | | |
| --- | --- | --- | --- |
| Authors | Articles | citation | average citation/publication |
| Parvizi. J | 170 | 7615 | 44.79 |
| Abdel. MP | 60 | 645 | 10.75 |
| Chen. AF | 59 | 2019 | 34.22 |
| Mont. MA | 58 | 1452 | 25.03 |
| Della-Valle. CJ | 57 | 2096 | 36.77 |
| Gehrke.T | 49 | 442 | 9.02 |
| Berry. DJ | 47 | 1685 | 35.85 |
| Tan. TL | 44 | 1880 | 42.73 |
| Schwarzkopf. R | 43 | 496 | 11.53 |
| Higuera. CA | 37 | 1079 | 29.16 |

**Supplementary Table 8 The top 10 authors with the highest number of publications**

| the 50 most frequently occurring author keywords | |
| --- | --- |
| keyword | occurrences |
| tha | 1017 |
| pji | 887 |
| arthroplasty | 423 |
| infection | 371 |
| complication | 330 |
| tka | 324 |
| revision | 265 |
| hip | 238 |
| total joint arthroplasty | 219 |
| hip arthroplasty | 210 |
| outcome | 147 |
| ssi | 129 |
| diagnosis | 104 |
| systematic review | 103 |
| risk factor | 100 |
| knee | 92 |
| revision tha | 92 |
| two-stage revision | 84 |
| dislocation | 79 |
| obesity | 67 |
| revision hip arthroplasty | 66 |
| knee arthroplasty | 59 |
| dair | 58 |
| hip fracture | 56 |
| biofilm | 52 |
| aseptic loosening | 50 |
| periprosthetic fracture | 50 |
| mortality | 48 |
| femoral neck fracture | 45 |
| readmission | 43 |
| antibiotic | 41 |
| crp | 39 |
| postoperative complications | 37 |
| osteoarthritis | 36 |
| treatment | 36 |
| direct anterior approach | 34 |
| orthopedic surgery | 33 |
| tranexamic acid | 32 |
| rheumatoid arthritis | 31 |
| prevention | 30 |
| staphylococcus aureus | 30 |
| synovial fluid | 29 |
| hemiarthroplasty | 28 |
| antibiotic prophylaxis | 27 |
| one-stage revision | 27 |
| dual mobility | 26 |
| spacer | 26 |
| transfusion | 25 |
| diabetes | 24 |
| epidemiology | 24 |
| sonication | 24 |
| acetabular defect | 23 |
| blood loss | 23 |
| cementless | 23 |
| instability | 23 |
| survival | 23 |
| surgery | 22 |
| length of stay | 21 |
| cost | 20 |
| osteomyelitis | 20 |
| septic arthritis | 20 |
| survivorship | 20 |

**Supplementary Table 9 the 50 most frequently occurring author keywords**
